# Geographical Origin Authentication of Wild Ginseng by Volatile and Non-Volatile Fingerprinting Across Growth Years

**DOI:** 10.3390/foods15132310

**Published:** 2026-06-29

**Authors:** Lili Cui, Rui Wang, Hongying Guo, Yuhe Ren, Ying Guo, Xinru Liu, Xuan Li, Meiling Jin, Jing Luo, Hui Zhao

**Affiliations:** Institute of Special Wild Economic Animal and Plant Science, Chinese Academy of Agricultural Sciences, Changchun 130112, China; cbscui@126.com (L.C.); 18744108018@163.com (R.W.); gerhyy@163.com (H.G.); renyuhe1123@163.com (Y.R.); 13756218145@163.com (Y.G.); rrg0319@163.com (X.L.); ndlixuan@163.com (X.L.); lovemeiling521@hotmail.com (M.J.); luojing01@caas.cn (J.L.)

**Keywords:** WG, geographical origin, HS-GC-IMS, ginsenosides, chemometrics

## Abstract

The chemical composition and perceived quality of wild ginseng (WG) are influenced by its geographical origin, yet clear chemical criteria for origin authentication remain lacking, and this problem is further complicated by the confounding effect of growth year. In this study, volatile and non-volatile fingerprints of 15- and 20-year-old WG from Huanren, Tonghua, and Ji’an (China) were characterized using HS-GC-IMS and HPLC. Partial least squares discriminant analysis (PLS-DA) models constructed separately for each growth-year group achieved complete origin separation of volatile fingerprints, with ten shared VIP markers identified. However, none maintained a consistent origin ranking across years, suggesting that the geographical signal may reside in the multivariate patterns. Ginsenoside profiling revealed a hierarchical candidate marker system: Rf, Rg1, Rc, Rb2 as robust candidate markers for Huanren, and Re as a 20-year-specific discriminator for Tonghua. The pervasive origin × growth year interaction observed across both volatile and non-volatile fractions indicates that growth-year-specific chemometric models are a necessity for reliable WG origin authentication. A practical two-step workflow—growth year determination followed by age-matched origin assignment—is proposed. These findings provide a scientific foundation for the geographical traceability and quality evaluation of WG.

## 1. Introduction

Ginseng (*Panax ginseng* C. A. Mey.), a perennial herb of the Araliaceae family, has been valued for its medicinal properties for over four millennia and occupies a prominent position in both traditional Chinese medicine and East Asian dietary culture [1]. With its recent classification as a new resource food, ginseng has gained expanded application in the modern food industry, being processed into teas, slices, powders, and incorporated into composite products such as beverages and pastries [2,3]. Among the various types of ginseng—categorized by cultivation method as original ecological, wild, forest-cultivated, and garden-cultivated—WG is the most prized. WG refers specifically to ginseng grown in natural mountain forest environments for over 15 years following artificial seeding [4]. Thriving at altitudes of 400–1000 m under prolonged environmental stresses including cold, shade, and nutrient competition, WG develops distinctive morphological traits and accumulates a rich array of bioactive substances, including ginsenosides, volatile oils, polysaccharides, and inorganic elements [5,6].

The quality and sensory characteristics of WG are intimately linked to its chemical composition. Its characteristic herbal and fresh aroma originates from volatile flavor compounds—primarily terpenes, alcohols, and aldehydes—whose composition directly influences the aromatic profile of ginseng-based food products. These volatile metabolites, together with ginsenosides, undergo systematic changes during plant development: ginsenoside content increases with growth year [7], total volatile oil content rises in young plants [8], and phytosterol levels are positively correlated with age [9]. Understanding how these compositional dynamics interact with geographical origin is essential for establishing a scientific basis for WG quality evaluation.

In China, WG is primarily distributed across the Changbai Mountain range and its remaining veins. To safeguard its regional quality and reputation, multiple geographical indication protections have been established, including GB/T 19506-2009 [10] for Jilin Changbai Mountain ginseng [11] and DB21/T 4252-2025 [12] for Huanren WG. Huanren County (Liaoning Province), Tonghua County, and Ji’an City (Jilin Province) represent the three core WG-producing areas. Although all three are situated within the Changbai Mountain remnants and share broadly similar macroclimatic conditions, they differ in microclimatic characteristics shaped by topography, latitude, and mountain barriers. Huanren, at the southern foot of the range, experiences a cool, humid continental climate (mean annual temperature 7.4 °C, precipitation 870.4 mm, frost-free period 135 days), with forests covering 79% of the county [13]. Ji’an, sheltered by the Laoling Mountains in the Yalu River valley, enjoys a semi-continental, semi-maritime climate with higher accumulated temperature (3650 °C), 800–1000 mm annual precipitation, and a longer frost-free period (150 days), with humus-rich dark brown forest soils (pH 5.5–6.5) [14]. Tonghua County, two-thirds mountainous, has a cooler and slightly drier climate (mean annual temperature 6.3 °C, precipitation 690–750 mm) with deep forest humus layers [15]. These microclimatic differences—particularly in temperature regime, growing season length, and soil conditions—may significantly influence the accumulation of secondary metabolites in WG [16], creating the chemical basis for origin differentiation.

However, despite the recognized influence of geographical origin on ginseng quality, clear chemical criteria for origin authentication of WG are still lacking, and the identification of origin-specific markers is further complicated by the confounding effect of growth year. Volatile flavor compounds, as products of secondary metabolism, respond sensitively to both environmental stress and developmental stage, potentially serving as “chemical fingerprints” of growth history. Ginsenosides, as the major pharmacologically active constituents, are closely tied to both efficacy and regional quality characteristics [17]. Systematic parallel analysis of these two chemical fractions therefore offers a comprehensive approach to understanding WG quality at the molecular level. HS-GC-IMS, which combines the separation power of GC with the sensitivity of ion mobility spectrometry, enables rapid, direct analysis of volatile and semi-volatile compounds without complex pretreatment and has shown notable advantages in food and traditional Chinese medicine authentication [18,19,20].

In this study, 15-year-old and 20-year-old WG from Huanren County, Tonghua County, and Ji’an City—three core origins within the Changbai Mountain range—were systematically analyzed. Volatile components were profiled by HS-GC-IMS with retention index-based identification and internal standard quantification, while seven major ginsenosides were determined by HPLC. The objectives were: (1) to characterize the volatile and non-volatile chemical fingerprints of WG from different origins, (2) to evaluate the influence of growth year and its interaction with origin on these chemical profiles, and (3) to screen characteristic markers and establish a multivariate strategy for geographical origin traceability. The results provide a scientific foundation for the quality evaluation, genuineness assessment, and resource development of WG.

## 2. Materials and Methods

### 2.1. Samples and Reagents

**Figure 1:**
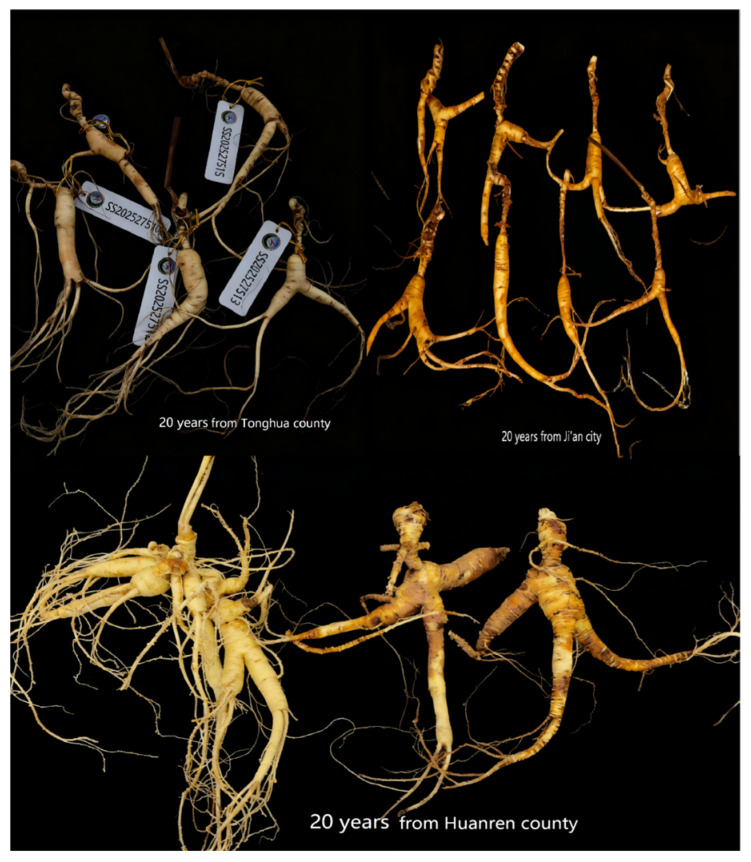
Partial wild ginseng samples collected from different origins during the experiment.

Samples were collected in August 2025 from three sampling sites: Huanren County (Liaoning Province), Tonghua County (Jilin Province), and Ji’an City (Jilin Province) (Figure 1). The geographic coordinates for Huanren are 41°23′23″ N, 125°25′44″ E, with an altitude of 443 m; for Tonghua County, 40°30′08″ N, 124°01′16″ E, altitude 455 m; and for Ji’an City, 41°46′09″ N, 137°42′50″ E, altitude 465 m. All three sites share the same vegetation type (coniferous and broad-leaved mixed forest) and slope aspect (northeast slope). A minimum of five independent and intact roots were collected from each sampling site, pooled to form a representative composite sample and labeled as gs10, gs18, gs19 (15-year-old, Huanren, Tonghua, Ji’an) and gs14, gs21, gs22 (20-year-old, Huanren, Tonghua, Ji’an). After washing, samples were dried at 50 °C, ground into powder (80 mesh), and stored at −20 °C until analysis.

Ginsenoside standards (Rg1, Re, Rf, Rb1, Rc, Rb2, Rd; purity > 98%) were purchased from Yuanye Bio-Technology Co., Ltd. (Shanghai, China). HPLC-grade methanol, acetonitrile and phosphoric acid were obtained from Merck (Darmstadt, Germany). GC-IMS calibration standards (C4–C9 n-ketones) and internal standard (2-methyl-3-heptanone, purity 99.5%) were purchased from Aladdin (Shanghai, China) and TanMo Reference Materials Company Ltd. (Changzhou, China), respectively.

### 2.2. GC-IMS Analysis

HS-GC-IMS analysis was performed using a FlavourSpec^®^ instrument (G.A.S. GmbH, Dortmund, Germany) equipped with a CTC PAL3 automatic headspace sampler (CTC Analytics AG, Zwingen, Switzerland) and an MXT-5 capillary column (15 m × 0.53 mm, 1.0 μm, Restek Corporation, Bellefonte, PA, USA), following the method of Zou et al. (2025) and Xiang et al. (2024) [18,19] with minor modifications. Sample (0.5000 g) was placed in a 20 mL headspace vial with 5 μL of internal standard (2500 μg/mL). Incubation was carried out at 70 °C for 20 min with agitation at 500 rpm. The injection volume was 500 μL, and the syringe temperature was 85 °C. The column temperature was maintained at 60 °C. High-purity N_2_ was used as carrier gas with a flow program: initial 2 mL/min for 2 min, increased to 10 mL/min in 8 min, then to 100 mL/min in 10 min, further to 150 mL/min in 10 min, and held for 30 min. The drift gas (N_2_) flow rate was 75 mL/min, drift tube temperature 45 °C, and electric field strength 500 V/cm. Compounds were tentatively identified by matching retention index (RI) calculated from n-ketone standards and drift time with databases—the NIST 2020 library and the IMS databases. Semi-quantification was performed using the internal standard method. The content of each target compound (mg/kg) was calculated according to the following formula:

$$W_{x}\;=\;\frac{C_{\mathrm{is}}\;\times {\;V}_{\mathrm{is}}}{1000\;\times \;m}\times \frac{A_{x}}{A_{\mathrm{is}}}$$

where Wx represents the content of the target compound, C_is_ is the concentration of the internal standard (µg/mL), Vis is the volume of internal standard added (µL), m is the sample weight (g), and Ax and Ais are the peak areas of the target compound and the internal standard, respectively.

### 2.3. HPLC Analysis of Ginsenosides

Sample powder (1.0000 g) was extracted with 25 mL of 75% methanol by ultrasonication for 30 min, then allowed to stand overnight, followed by another 60 min of ultrasonication. After filtration (0.22 μm), the extract was analyzed by an Acquity UPLC H-Class system (Waters, Milford, MA, USA) with a BEH C18 column (1.7 μm, 2.1 × 100 mm, Waters, USA). Mobile phase consisted of (A) acetonitrile and (B) water with 0.1% phosphoric acid, at a flow rate of 0.4 mL/min and column temperature of 45 °C. The gradient elution program was as follows: 0–4.81 min, 19% A; 4.81–7.31 min, 19–21% A; 7.31–10.31 min, 21–28% A; 10.31–16.31 min, 28–31% A; 16.31–21.31 min, 31–38.5% A; 21.31–22.00 min, 38.5–90% A; 22.00–25.00 min, 90% A; 25.00–25.50 min, 90–19% A; 25.50–27.50 min, 19% A [21]. Detection wavelength was 203 nm. Ginsenosides were quantified using external calibration curves. Ginsenoside content was calculated according to the following formula:

$$X(\%)\;=\;\frac{C_{s}\;\times \;V}{m\;\times \;10}$$

where X represents ginsenoside content (%), C_s_ is the measured concentration of ginsenoside (mg/mL), V is the total volume of sample extract solution (mL), and m is the sample weight (g).

### 2.4. Determination of Total Ginsenosides

Total ginsenosides were determined by the vanillin–sulfuric acid colorimetric method using an ultraviolet-visible (UV-Vis) spectrophotometer, with ginsenoside Re as the reference standard, in accordance with the method prescribed in GB/T 18765-2015 [22].

#### 2.4.1. Preparation of Standard Curve

A stock solution of ginsenoside Re (1.0 mg/mL) was prepared in methanol. Aliquots of 0, 20, 40, 60, 80, 100, and 120 μL of the stock solution were transferred into glass-stoppered test tubes and evaporated to dryness in a water bath. To each tube, 0.5 mL of freshly prepared 8% (*w*/*v*) vanillin–ethanol solution and 5 mL of 72% (*v*/*v*) sulfuric acid solution were added. The mixture was thoroughly shaken, incubated at 60 °C in a water bath for 10 min, cooled immediately in an ice water bath for 10 min, and shaken again. The absorbance was measured at 544 nm against a reagent blank, and the standard curve was constructed by plotting absorbance versus Re concentration.

#### 2.4.2. Sample Determination

The sample solution was prepared as follows. Approximately 1 g of WG powder was accurately weighed, wrapped in neutral filter paper, and defatted with diethyl ether in a Soxhlet extractor under reflux for 1 h. After discarding the ether extract, the defatted sample was extracted with methanol in a Soxhlet extractor under reflux for at least 3 h. The methanol extract was concentrated and the residue was dissolved in 30–40 mL of distilled water and extracted with water-saturated n-butanol (30 mL × 4). The combined butanol extracts were evaporated to dryness, and the residue was redissolved in methanol and made up to a final volume of 10 mL. A 20 μL aliquot of this solution was evaporated to dryness and subjected to the same colorimetric procedure as described for the standard curve. The absorbance was measured at 544 nm, and the total ginsenoside content was calculated from the standard curve and expressed as milligrams of ginsenoside Re equivalents per gram of dry weight (mg/g DW). All determinations were performed in triplicate.

### 2.5. Data Processing and Statistical Analysis

GC-IMS data were processed using VOCal software (version 0.4.03, G.A.S. GmbH, Dortmund, Germany). One-way analysis of variance (ANOVA) followed by Tukey’s post hoc test (*p* < 0.05) was performed using SPSS 26.0. Partial least squares discriminant analysis (PLS-DA) and variable importance in projection (VIP) were conducted with SIMCA 14.1 (Umetrics). Hierarchical clustering heatmaps were generated using Origin 2025b. All experiments were conducted in triplicate, and the data are presented as the mean ± Standard Deviation (SD).

## 3. Results

### 3.1. Identification and Classification of Volatile Compounds

A total of 67 volatile compounds were tentatively identified in WG samples from the three origins, belonging to terpenes, ketones, aldehydes, esters, alcohols, pyrazines, organic acids, and others [23] (Table 1). These compounds have characteristic odors, such as the citrus- and woody-like notes of terpenes, the grassy and fruity notes of aldehydes, and the roasted and nutty notes of pyrazines, which collectively contribute to the characteristic medicinal aroma of WG.

**Figure 2:**
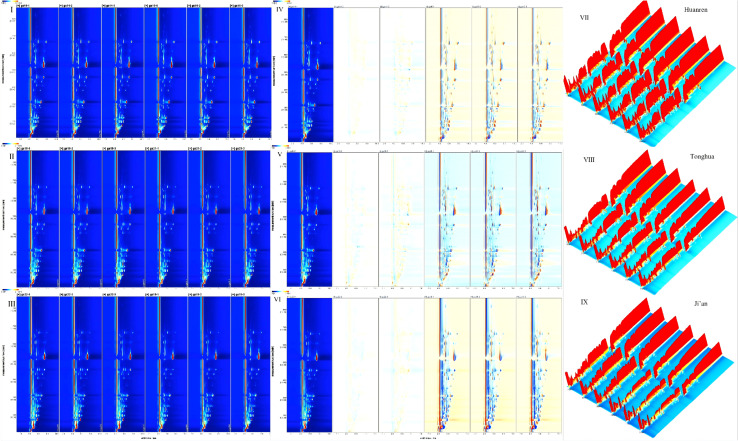
Representative GC-IMS spectra of wild ginseng from different origins. Note: (**I**–**III**) is two-dimensional, (**IV**–**VI**) is a comparative map, (**VII**–**IX**) is three-dimensional.

**Figure 3:**
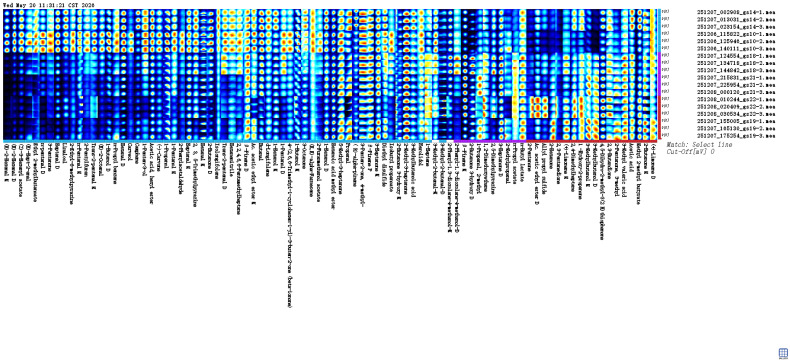
Gallery plot of volatile fingerprints of wild ginseng from three origins. Note: red is higher content, blue is lower content.

The three-dimensional, two-dimensional, and differential GC-IMS spectra (Figure 2) showed clear differences among the three origins. The signal intensities intuitively indicated that WG is rich in volatile flavor compounds, with peak positions and intensities varying significantly across origins. The fingerprint demonstrated that samples from the same origin clustered together, while distinct patterns were observed across origins. The fingerprint gallery plot (Figure 3) visually showed high spectral overlap among samples from the same origin but significant differences among different origins, further verifying the regulatory effect of origins on volatile components of WG.

### 3.2. Origin Differences in Volatile Compounds

#### 3.2.1. Multivariate Classification Models for 15-Year and 20-Year Samples

**Figure 4:**
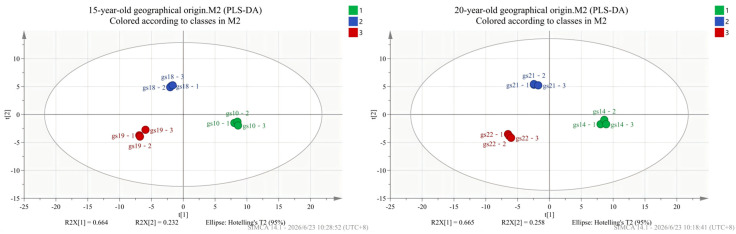
PLS-DA score plots for samples from different geographical origins collected in different growth years.

**Figure 5:**
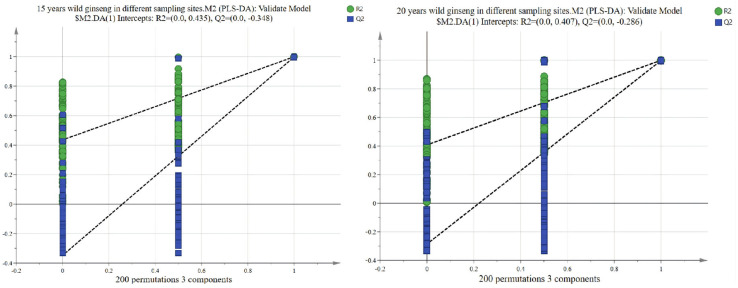
Permutation test validation confirming the robustness and non-overfitting of PLS-DA models for geographical origin wild ginseng across different growth years.

To explicitly evaluate the ability of volatile components to discriminate geographical origins, we constructed PLS-DA models for 15-year-old and 20-year-old WG separately, with origin as the dependent variable (Y) and volatile compound contents as independent variables (X). The score plots (Figure 4) showed that samples from Huanren, Tonghua, and Ji’an exhibited a clear separation trend along the first two latent variables in both age cohorts, with these two latent variables collectively explaining 89.6–92.3% of the total variance. Permutation tests with 200 iterations (Figure 5) confirmed the robustness of both models: the R^2^ intercepts were 0.435 (15-year-old) and 0.407 (20-year-old), while the Q^2^ intercepts were −0.348 and −0.286, respectively, indicating no overfitting and validating the statistical significance of the models (*p* < 0.05). These results demonstrate that, regardless of the growth year, the volatile fingerprint of WG contains stable and robust information for geographical origin discrimination.

#### 3.2.2. Identification of Shared Volatile Markers and the Pervasive Origin × Year Interaction

**Figure 6:**
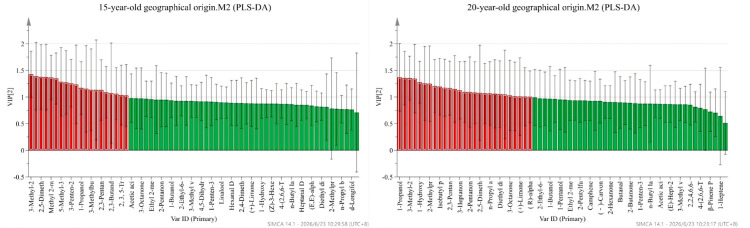
Volatile markers with VIP > 1.0 contributing to origin discrimination in 15- and 20-year wild ginseng. Note: Red bars denote variables with VIP values > 1.0, while green bars represent those with VIP values < 1.0.

**Figure 7:**
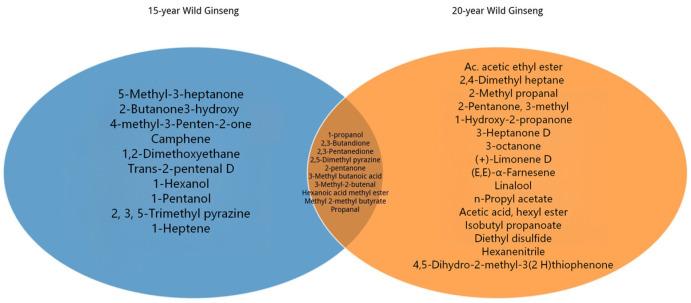
Overlap of characteristic volatile markers (VIP > 1.0) between PLS-DA models constructed for 15- and 20-year wild ginseng.

VIP values were used to screen characteristic markers contributing to origin separation, with a threshold of VIP > 1.0 (Figure 6). Twenty markers were identified in the 15-year model and twenty-six in the 20-year model, among which ten compounds were shared across both growth years (Figure 7): 3-methyl-2-butenal, propanal, 2,5-dimethyl pyrazine, 2,3-butandione, 2-pentanone, 2,3-pentanedione, 1-propanol,2-methyl, methyl 2-methyl butyrate, hexanoic acid methyl ester, and 3-methyl butanoic acid. These ten compounds were regarded as the core volatile fingerprints for origin discrimination.

Notably, when their individual contents were compared across origins by univariate analysis, none of these ten markers maintained a consistent origin ranking across the two growth-year groups (Table 2). For example, 2-pentanone was the highest in Tonghua for 15-year-old ginseng (0.28 ± 0.015 mg/kg) but became the highest in Ji’an for 20-year-old ginseng (0.89 ± 0.016 mg/kg), exhibiting a complete reversal. Hexanoic acid methyl ester ranked Huanren > Ji’an > Tonghua at 15 years but shifted to Huanren > Tonghua > Ji’an at 20 years. 2,5-Dimethyl pyrazine displayed significant origin differences at 15 years (Tonghua > Huanren > Ji’an) yet no differences at 20 years, while 1-propanol,2-methyl was highest in Ji’an at 15 years but highest in Tonghua at 20 years. The complete absence of any marker with a stable origin ranking indicates that the volatile secondary metabolite profiles undergo a systematic reconfiguration between 15 and 20 years of growth, with the biosynthetic trajectories of each origin diverging or converging in a compound-specific manner.

Despite this striking year-dependent variability at the individual compound level, the PLS-DA models achieved complete (100%) origin separation for both growth-year groups.

This apparent paradox suggests that the geographical origin signal may be primarily encoded in the multivariate covariance structure among volatile markers, rather than in the absolute abundance of any single compound. However, direct evidence for a stable, origin-specific covariance pattern requires further investigation, such as correlation network analysis across multiple years and locations.

#### 3.2.3. Geographical Origin Signal Resides in Multivariate Covariance Structure Rather than Single Markers

The absence of any single compound with a consistent origin ranking across growth years underscores a crucial point: PLS-DA does not rely on individual compound thresholds but learns the characteristic ratios, combinations, and interrelationships that define each origin’s chemical space. In practical application, an unknown sample is assigned to an origin based on its projection within the latent variable space of the trained model, not by comparison to univariate benchmarks.

This principle is analogous to facial recognition algorithms that identify individuals based on the relative spatial configuration of facial features rather than on the absolute dimensions of any single feature.

The necessity of such multivariate approaches, rather than single-marker methods, is the norm in geographical origin studies of complex biological samples, where environmental and ontogenetic interactions preclude the existence of universally invariant markers. Consequently, any origin authentication strategy relying on fixed concentration thresholds of a few individual compounds would be fundamentally unreliable across different harvest ages.

#### 3.2.4. Convergence Across Chemical Fractions: The Necessity of Growth-Year-Specific Multivariate Modeling

**Figure 8:**
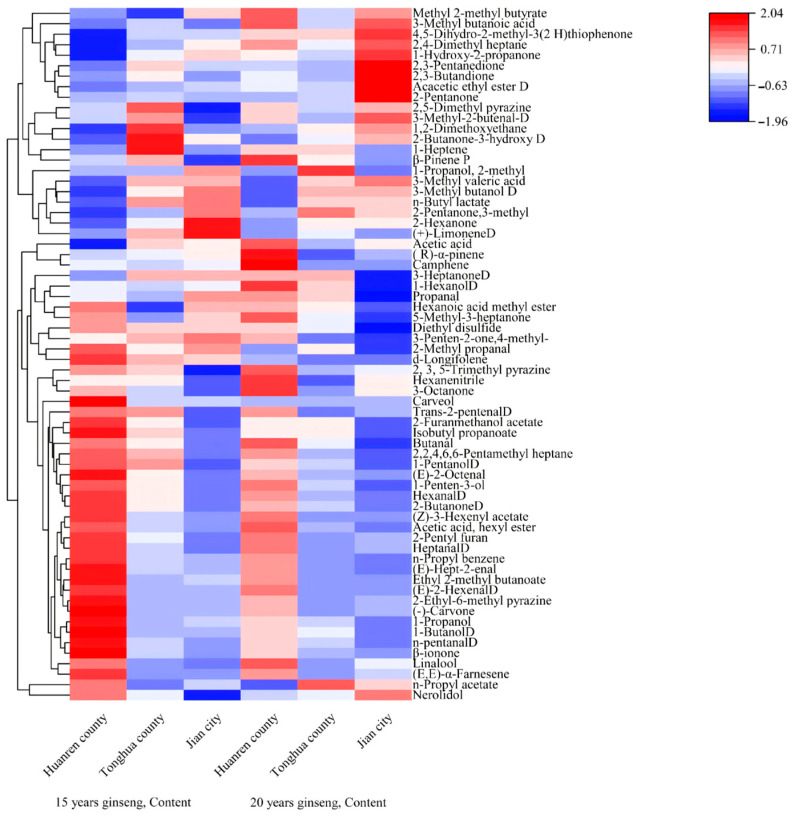
Heatmap of volatile flavor compounds in wild ginseng samples. Note: The color gradient, red = high abundance, blue = low abundance.

The pronounced origin × growth year interactions demonstrated above carry direct implications for the practical authentication of WG. The volatile metabolite heatmap (Figure 8) provides a comprehensive visual summary of these interactions. The color-coded abundance matrix, combined with hierarchical clustering, reveals two important patterns: first, samples are primarily grouped by origin, confirming that the geographical signal dominates over growth-year variation; second, within each origin cluster, 15-year and 20-year samples form distinct subclusters, visually reinforcing the year-dependent metabolic shifts identified in the preceding analyses. Specific accumulation patterns are readily discernible; for instance, certain esters and terpenoids are enriched in Huanren samples at both growth stages, whereas particular aldehydes and alcohols are preferentially accumulated in Ji’an samples only at 20 years.

These observations have practical consequences. Because the volatile fingerprint of a 15-year-old WG differs markedly from that of a 20-year-old WG even when collected from the same origin, a universal model trained on mixed-age samples would inevitably suffer from reduced discriminatory specificity. In contrast, PLS-DA models achieved complete (100%) separation of the three origins within each growth year. We therefore propose a two-step authentication workflow for real-world application: (1) the growth year is first determined, either by traditional morphological inspection (e.g., rhizome scar counting) or by a dedicated chemical model trained for age prediction; (2) the sample is then assigned to its geographical origin using the corresponding age-matched PLS-DA model. This stratified strategy ensures that the discriminant model operates within the appropriate chemical context, circumventing the confounding effect of year-dependent metabolic shifts.

The heatmap further corroborates the necessity of this stratified approach: as shown by the color gradients, no single volatile compound exhibits consistent origin-specific enrichment across both ages. The origin-specific signals are embedded in multivariate patterns that evolve over the plant’s lifespan, reinforcing the conclusion that only a combined strategy—age-stratified sampling coupled with multivariate pattern recognition—can deliver reliable geographical traceability. This approach respects the biological reality that secondary metabolite profiles change as WG matures, and it converts the challenge of year-dependency from an analytical obstacle into a manageable component of the authentication framework.

### 3.3. Ginsenoside Content Analysis

#### 3.3.1. Ginsenoside Compositional Variation and Origin-Specific Clustering

**Figure 9:**
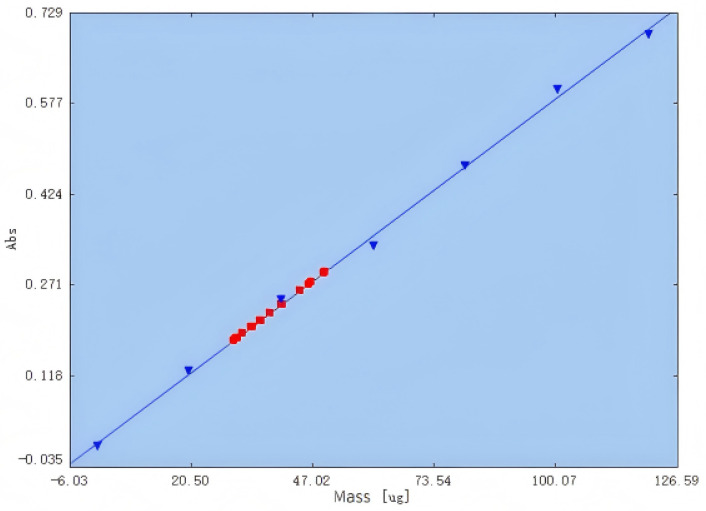
Standard curve of ginsenoside Re for total ginsenoside determination by the vanillin–sulfuric acid colorimetric method. Note: The regression equation: y = 0.0058χ + 0.0045, correlation coefficient R^2^ = 0.9998. Note: blue triangles represent concentration points of the calibration curve, and red circles stand for test samples.

**Figure 10:**
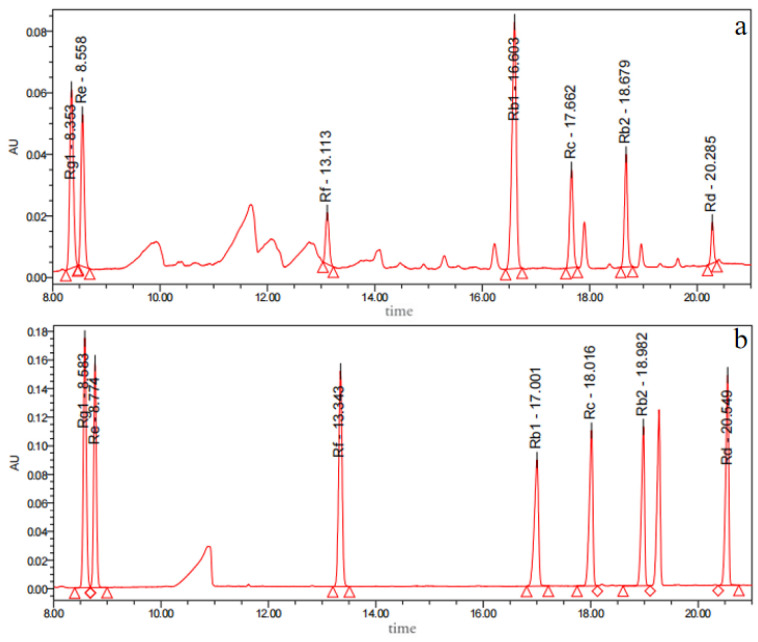
Representative HPLC chromatograms of ginsenosides in wild ginseng samples. (**a**) Wild ginseng sample chromatogram; (**b**) Mixed ginsenoside standard chromatogram. Triangles under peaks mark retention time positions of seven ginsenosides (Rg1, Re, Rf, Rb1, Rc, Rb2, Rd). Peak labels are formatted as “ginsenoside name abbreviation–retention time (min)”.

**Figure 11:**
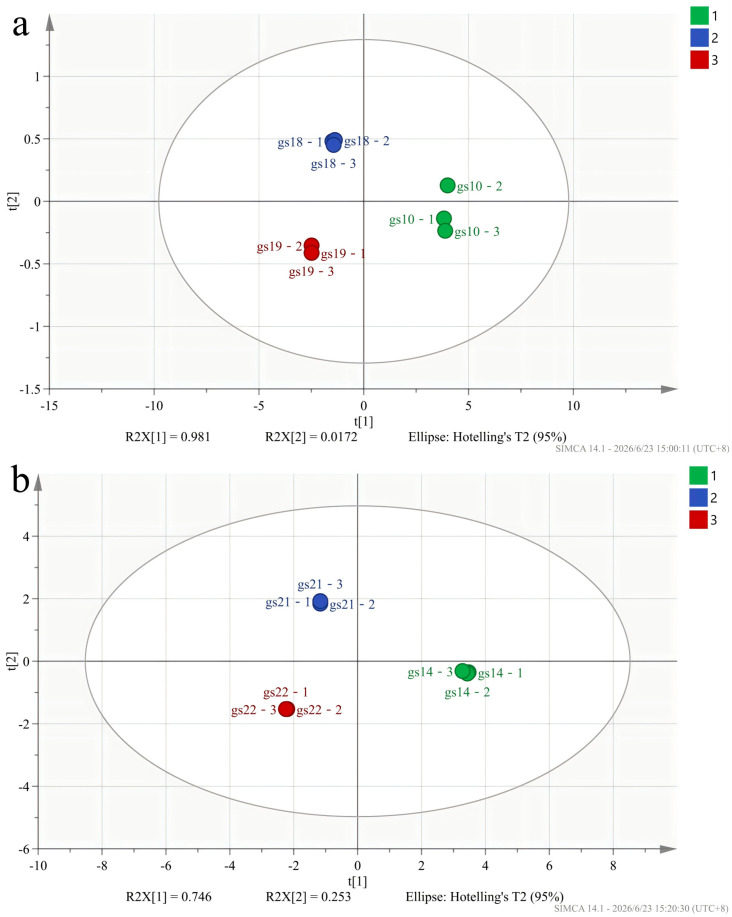
PLS-DA score plots of ginsenoside composition in wild ginseng from different geographical origins. Note: (**a**): 15-year-old wild ginseng; (**b**): 20-year-old wild ginseng.

**Figure 12:**
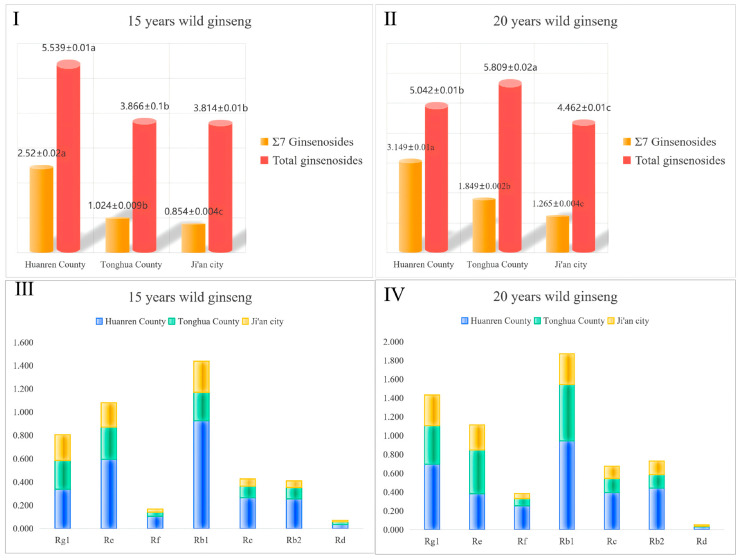
Ginsenoside profiles of wild ginseng from three geographical origins at two growth years. Note: (**I**,**II**) Total ginsenosides determined by colorimetric assay and the sum of seven individually quantified ginsenosides (Rg1, Re, Rf, Rb1, Rc, Rb2, Rd) by HPLC. Data are presented as mean ± SD (n = 3). Letters a,b,c indicate significant differences between different geographical origins at the same growth year (*p* < 0.05). (**III**,**IV**) Compositional profiles of the seven ginsenosides in each group, shown as stacked bars. Different colors represent individual ginsenosides as indicated in the legend.

To comprehensively characterize origin-related differences in ginsenoside profiles, total ginsenosides (Figure 9), the sum of seven individually quantified ginsenosides (∑7 ginsenosides) (Figure 10), and the compositional distribution of individual ginsenosides were determined for WG samples from Huanren, Tonghua, and Ji’an at both 15 and 20 years of growth. The overall clustering patterns were evaluated by partial least squares discriminant analysis (PLS-DA), as shown in Figure 11 and Figure 12.

The PLS-DA score plots demonstrated clear, origin-specific clustering of WG samples at both growth years, confirming that ginsenoside composition constitutes a reliable chemical fingerprint for geographical origin discrimination. For 15-year-old ginseng, the first latent variable (LV1) accounted for 98.12% of the total variance, indicating that origin differentiation was dominated by a single chemical dimension. Huanren samples were tightly clustered on the positive side of LV1 (t [1] ≈ 4), whereas Ji’an and Tonghua samples were located on the negative side (t [1] ≈ −2 to −3), with the three origins exhibiting completely non-overlapping clusters. For 20-year-old ginseng, LV1 and LV2 explained 71.7% and 28.3% of the variance, respectively. The origin separation evolved into a multidimensional pattern: Huanren samples remained on the positive side of LV1, while Ji’an (t [2] = −1.56895) and Tonghua (t [2] = 1.88067) samples achieved complete mutual separation along LV2, with substantially enlarged inter-origin distances compared with the 15-year cohort.

Model validation confirmed the robustness of both PLS-DA models. For the 15-year model, R^2^Y = 0.999 and Q^2^ = 0.984; for the 20-year model, R^2^Y = 1.0 and Q^2^ = 1.0. Permutation tests with 200 iterations yielded R^2^Y intercepts of 0.321 and 0.599, and Q^2^ intercepts of −0.209 and −0.043, respectively (Supplementary Figure S1), confirming that neither model suffered from overfitting and that the observed origin separations were statistically significant.

The quantitative profiles underlying these multivariate patterns revealed distinct origin-specific accumulation characteristics. In 15-year-old ginseng, Huanren samples exhibited significantly higher total ginsenosides (5.539%) and Σ7 ginsenosides (2.52%) than Tonghua (3.866% and 1.02%) and Ji’an (3.814% and 0.854%). This superiority was driven by uniformly higher accumulation of all major ginsenosides—Rb1 (0.926%), Re (0.596%), Rg1 (0.337%), and others—rather than by the enrichment of any single compound.

In 20-year-old ginseng, a pronounced origin × growth year interaction emerged. The age-dependent trajectory of total ginsenosides diverged among origins: Tonghua (3.866%→5.809%) and Ji’an (3.814%→4.462%) showed marked increases, whereas Huanren (5.539%→5.042%) exhibited a slight decline. Compositionally, Huanren retained its absolute advantage in Σ7 ginsenosides (3.15%), with the highest Rb1 (0.944%) and Rg1 (0.698%) levels. Tonghua was characterized by a specific enrichment of Re (0.462%), while Ji’an displayed a distinct accumulation pattern of Rd, which increased from 0.013% to 0.015%—in sharp contrast to the declining trends observed in Huanren (0.035%→0.031%) and Tonghua (0.022%→0.007%).

Taken together, these results demonstrate that the cumulative effect of geographical environment on ginsenoside biosynthesis is progressively amplified with increasing growth year. The chemical differentiation among origins evolves from a unidimensional, overall-content-driven distinction at 15 years into a multidimensional, composition-specific signature at 20 years.

#### 3.3.2. Association Between Individual Ginsenosides and Origins

**Figure 13:**
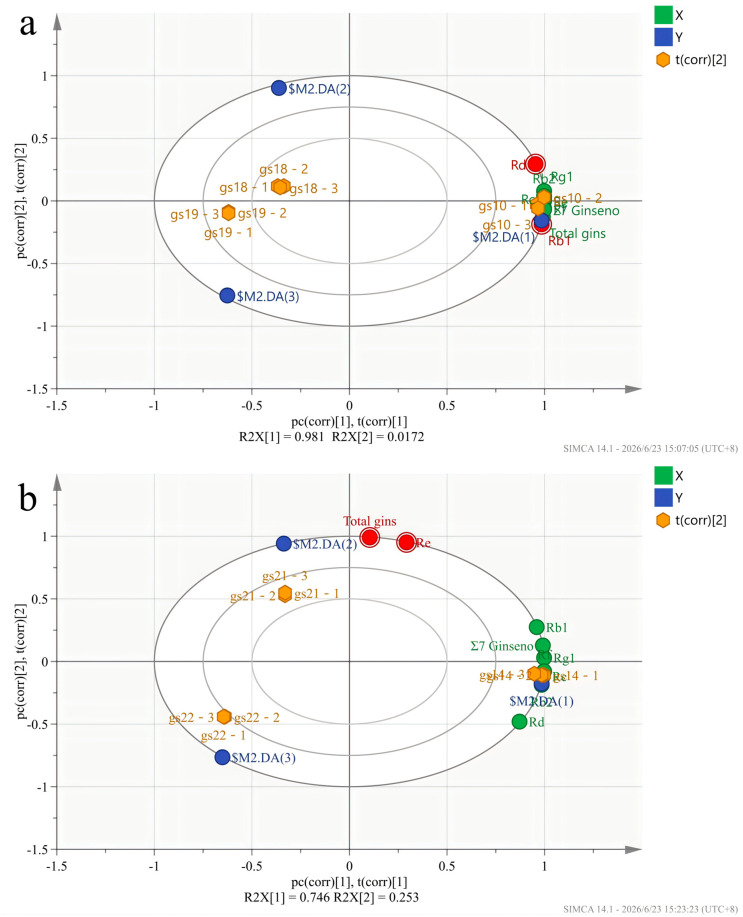
PLS-DA biplots showing the correlation between ginsenoside components and geographical origins. (**a**) 15-year-old wild ginseng; (**b**) 20-year-old wild ginseng.

To identify the specific ginsenosides responsible for the observed origin separation, PLS-DA biplots were constructed, simultaneously displaying sample scores and variable loadings (Figure 13).

In the 15-year-old model (Figure 13a), all detected ginsenosides (Rg1, Rb1, Re, Rf, Rb2, Rd) and total ginsenosides were concentrated on the positive side of LV1, exhibiting strong positive correlations with the Huanren sample cluster. Neither Ji’an nor Tonghua samples showed significant positive associations with any individual ginsenoside. This indicates that the origin difference at 15 years is essentially a difference in overall ginsenoside abundance: Huanren 15-year-old WG is comprehensively superior to its Ji’an and Tonghua counterparts across all measured saponin constituents.

The 20-year-old model (Figure 13b) revealed a more nuanced and informative pattern. Most ginsenosides (Rg1, Rb1, Rf, Rd, Rb2) and total ginsenosides remained clustered on the positive side of LV1 and were strongly associated with Huanren samples, confirming that Huanren 20-year-old ginseng retains its dominant advantage in overall ginsenoside content. Critically, Re and total ginsenosides uniquely occupied the positive side of LV2, exhibiting a strong positive correlation with Tonghua samples. This reveals a key origin-specific biosynthetic divergence emerging with extended growth: Tonghua 20-year-old WG is characterized by the highest total ginsenoside content, followed by Huanren, with a distinctive enrichment in Re. Ji’an samples, in contrast, showed no significant positive association with any ginsenoside, indicating the lowest overall saponin content among the three origins.

#### 3.3.3. Quantitative Screening of Origin-Specific Ginsenoside Markers

**Figure 14:**
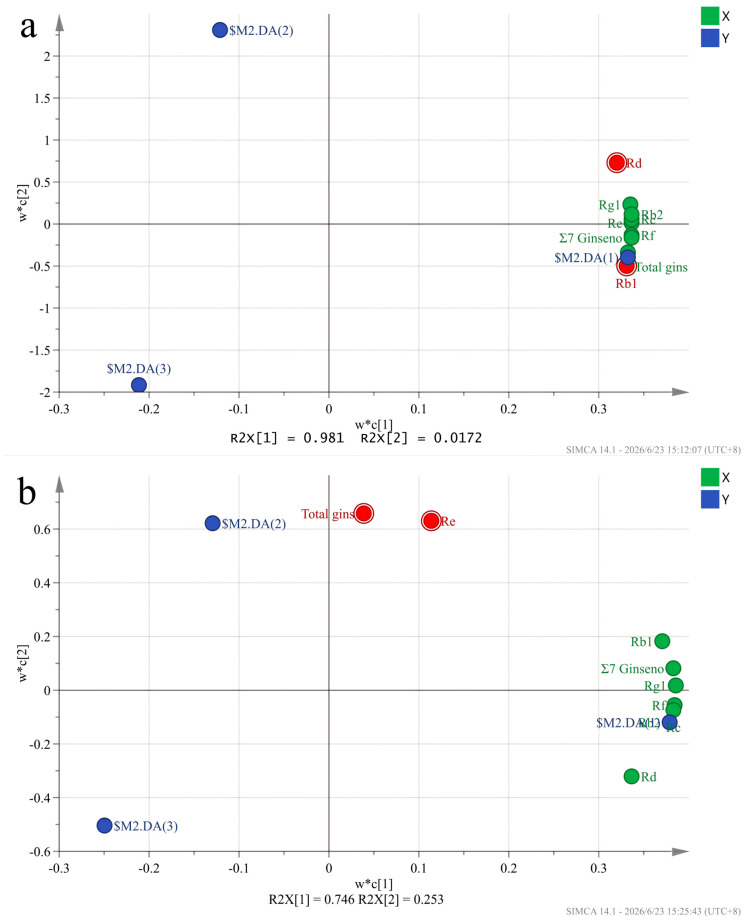
PLS-DA loading scatter plots for screening geographical origin-specific ginsenoside markers. Note: (**a**) 15-year-old WG; (**b**) 20-year-old WG.

To quantitatively assess the contribution of each ginsenoside to origin discrimination and to screen statistically robust markers, loading scatter plots were generated (Figure 14). The absolute value of the loading weight (w*c) reflects the magnitude of a variable’s contribution to origin separation along a given latent variable, while the sign indicates the direction of its correlation with each origin. For the 20-year model, both w*c [1] and w*c [2] were examined, corresponding to the two-dimensional origin separation revealed by the score plot (Figure 11b). For 15-year-old wild ginseng, all detected ginsenosides and total ginsenosides were located on the positive side of w*c [1], with loading values exceeding 0.3400, indicating that each contributed substantially to origin differentiation. The order of contribution was: Re (w*c [1] = 0.3573) > Rb2 (w*c [1] = 0.3570) > Rf (w*c [1] = 0.3565) > Rg1 (w*c [1] = 0.3561) > total ginsenosides (w*c [1] = 0.3530) > Rb1 (w*c [1] = 0.3504) > Rd (w*c [1] = 0.3408). The uniformly high and narrowly distributed loading values are consistent with the unidimensional separation observed in the score plot: at 15 years, all ginsenosides were positively associated with Huanren samples, and the origin difference was driven by a comprehensive abundance advantage rather than by the specific enrichment of any single compound.

For 20-year-old wild ginseng, the loading plot exhibited a clear two-dimensional marker distribution, reflecting the multivariate origin differentiation observed in the score plot. 1. LV1-direction universal markers—six ginsenosides exhibited w*c [1] values exceeding 0.3700: Rf (w*c [1] = 0.4168, w*c [2] = −0.0307), Rg1 (w*c [1] = 0.4167, w*c [2] = −0.0432), Rc (w*c [1] = 0.4163, w*c [2] = −0.0526), Rb2 (w*c [1] = 0.4131, w*c [2] = −0.0967), Rb1 (w*c [1] = 0.3973, w*c [2] = −0.2051), and Rd (w*c [1] = 0.3718, w*c [2] = −0.3014). Their high positive loadings on LV1 and near-zero or moderately negative loadings on LV2 establish them as robust universal markers for discriminating Huanren from Ji’an and Tonghua. Notably, Rf, Rg1, Rc, and Rb2 displayed the most favorable marker profiles, with w*c [1] > 0.41 and w*c [2] close to zero, indicating strong and highly specific association with Huanren origin. Rd showed a moderate negative loading on LV2 (w*c [2] = −0.30141), suggesting a partial contribution to the Ji’an–Tonghua discrimination along LV2, albeit secondary to its dominant role on LV1. 2. LV2-direction age-specific markers—Re (w*c [1] = 0.10866, w*c [2] = 0.64157) and total ginsenosides (w*c [1] = 0.02760, w*c [2] = 0.60347) displayed a strikingly distinct loading profile: exceptionally high positive loadings on LV2 coupled with negligible loadings on LV1. This unique pattern makes them the sole and definitive markers for discriminating Ji’an from Tonghua at 20 years. The near-zero w*c [1] values indicate that neither Re nor total ginsenosides contributed meaningfully to the Huanren-versus-others separation; their discriminatory power is entirely specific to the LV2 dimension, which captures the divergence between Ji’an and Tonghua that emerges only with extended growth. This result aligns precisely with the biplot analysis (Figure 13b), where Re and total ginsenosides uniquely occupied the positive side of LV2 and were strongly correlated with Tonghua samples.

Rf, Rg1, Rc, and Rb2 constitute the most robust candidate core markers for discriminating Huanren from the other two origins, displaying high LV1 loadings with minimal LV2 interference across growth years. Rb1 and Rd serve as additional candidate markers, though with moderate LV2 involvement at 20 years. Re functions as a candidate age-specific marker whose discriminating power is entirely confined to the LV2 dimension at 20 years, enabling effective differentiation between Ji’an and Tonghua origins that remain chemically indistinguishable at the younger growth stage.

## 4. Discussion

This study systematically compared the volatile (HS-GC-IMS) and non-volatile chemical fingerprints—the latter comprising seven ginsenoside monomers quantified by HPLC and total ginsenosides determined by the vanillin–perchloric acid colorimetric method—of wild ginseng from three northeastern Chinese origins (Huanren, Tonghua, and Ji’an) at two critical harvest ages (15 and 20 years), with the goal of establishing a robust geographical traceability framework. By analyzing the two chemical fractions through parallel yet methodologically differentiated chemometric approaches, several convergent and complementary findings emerged.

### 4.1. Volatile Compound Profiles of Wild Ginseng from Different Origins

Beyond the chemometric separation demonstrated by the PLS-DA models, the volatile composition of WG exhibited clear origin-specific qualitative characteristics. Huanren samples were distinguished by high contents of terpenes (nerolidol, d-longifolene) and aldehydes (hexanal, heptanal), Ji’an samples by the enrichment of esters (ethyl acetate, methyl 2-methylbutyrate), and Tonghua samples by relatively higher levels of pyrazines (2,5-dimethylpyrazine). These compositional biases carry sensory implications: terpenes and aldehydes typically contribute woody, grassy, and fatty notes [24,25,26], whereas esters impart fruity and sweet aromas [27], and pyrazines are associated with roasted and nutty characters. Consequently, the volatile fingerprints identified here may also underlie perceptible differences in the aromatic quality of WG from different origins.

From a biosynthetic perspective, the observed origin-specific accumulation patterns are consistent with known environmental influences on secondary metabolism. Terpenes, aldehydes, alcohols, esters, and pyrazines are derived from distinct biosynthetic routes [28,29,30]. However, whether the regional differences observed here—e.g., the terpene- and aldehyde-dominated profile of Huanren ginseng versus the ester-rich profile of Ji’an ginseng—stem from altered pathway flux, genetic variation, or rhizosphere microbial interactions cannot be determined from the current data. These mechanistic interpretations remain speculative and should be tested in future studies using targeted enzyme activity assays, transcriptomics, or soil microbiome analysis.

Notably, the three sampling sites are all located within the Changbai Mountain range, sharing broadly similar macro-environmental conditions of altitude, slope orientation, and forest vegetation type. The observation of significant chemical differentiation under such macro-environmental similarity points to the decisive role of micro-environmental factors—including soil physicochemical properties, trace element availability, and rhizosphere microbial community composition—in shaping the secondary metabolome of WG [7,8]. These findings thus provide a chemical rationale for the geographical indication status of WG products and align with growing evidence that micro-environmental heterogeneity, even at local scales, can drive meaningful phytochemical divergence in medicinal plants.

### 4.2. Volatiles and Ginsenosides: Why Growth Year Matters for Origin Authentication

The centrality of age-stratified multivariate modeling. The most consistent finding across both the volatile and ginsenoside datasets was that geographical origin can be discriminated with high confidence, but only when the statistical model accounts for the growth year. In the volatile fraction, PLS-DA models achieved complete origin separation within each age cohort, yet none of the ten shared VIP markers maintained a stable origin ranking across 15 and 20 years. In the ginsenoside fraction, the PLS-DA score plots likewise yielded clear origin-specific clustering, with the model explanatory power evolving from a unidimensional structure (LV1 = 98.12%) at 15 years to a multidimensional structure (LV1 + LV2 = 100%) at 20 years. This parallel behavior—robust multivariate separation coexisting with pervasive year-dependency at the individual marker level—was observed in two chemically orthogonal fractions (terpenoid volatiles vs. triterpenoid saponins), strongly suggesting that the origin × growth year interaction is an intrinsic feature of secondary metabolism in WG rather than an artifact of a particular analytical platform.

Earlier investigations have laid a solid foundation for WG chemistry and age-related differences. Regarding ginsenosides, Yu et al. [31] reported that the ratios of total ginsenosides, Re and Rb1 to Rg1 gradually increase with age in WG, suggesting that these ratios could serve as age indicators. Recent Korean studies on wild-simulated ginseng (WSG) further explored the relationship between growth conditions, harvest time and ginsenoside accumulation. Kim et al. [32] found that 13-year-old wild-simulated ginseng had significantly higher contents of Rb1, Rb2, Rc, Rd, Re, Rf and Rg1 than 7-year-old plants. Yun et al. [33] reported that total ginsenoside content peaked in July, largely due to increased levels of F2, Rb2, Rb3, Re and Rg1 in the aerial part. Dai et al. [34] observed that protopanaxadiol-type (PPD) ginsenosides increase more rapidly with age than protopanaxatriol-type (PPT) ones. For volatiles, Zhu et al. [35] compared 5-year cultivated ginseng and 30-year WG, identifying 69 volatile compounds and showing that WG is richer in bicyclic and tricyclic sesquiterpenes. For geographical origin tracing, Xing et al. [36] developed near-infrared (NIR) models for WG from the city of Jilin province and the Huanren Manchu Autonomous County of Liaoning province, achieving recognition rates of 95–98%.

Despite these valuable contributions, two critical gaps remain. First, the single-dimension focus: most studies examined either ginsenosides or volatiles separately, lacking a parallel comparison of both chemical fractions in the same set of samples. Second, the absence of systematic investigation of the origin × year interaction: while age-related changes and between-origin differences have been reported separately, whether the chemical differentiation among origins remains stable or changes with growth year has never been directly examined. NIR-based methods, although rapid, do not resolve the confounding effect of age on origin discrimination.

How the present study complements previous work: the current study directly addresses the above gaps. First, by simultaneously analyzing volatiles and ginsenosides in the same samples, we show for the first time that both chemical fractions exhibit the same age-dependent behavior. Second, by comparing 15-year and 20-year WG from three origins, we provide the first explicit evidence that the chemical differentiation among origins is not age-invariant. Third, based on this interaction, we propose an age-stratified authentication strategy (detailed in Section 4.4) that converts growth year from a confounding factor into an integral component of the authentication workflow. These findings extend them by revealing a previously unrecognized layer of complexity—the interplay between origin and age—and offer a more refined methodological framework for WG geographical traceability.

### 4.3. Volatiles and Ginsenosides: Two Ways of Encoding Origin Information

Although both volatiles and ginsenosides show a strong dependence on growth year, they differ fundamentally in how they encode geographical origin. This difference has direct implications for authentication strategy.

#### 4.3.1. Volatiles: Rely on the Overall Pattern, Not on Any Single Compound

Between 15 and 20 years of growth, no single volatile marker maintains a stable origin ranking. A compound that is highest in Huanren at 15 years may become highest in Ji’an at 20 years. Therefore, it is impossible to use a simple rule such as “high content of compound X indicates origin Y”. The only reliable approach is to analyze the entire set of volatiles as a holistic pattern using multivariate models such as PLS-DA. This is similar to facial recognition-identification depends not on the size of the eyes or nose alone, but on the overall configuration of facial features.

#### 4.3.2. Ginsenosides: Rely on a Hierarchical Set of Candidate Markers

In contrast, ginsenoside profiles are more structured, and several regular patterns can be distilled into candidate markers:

For Huanren origin: At both 15 and 20 years, the four ginsenosides Rf, Rg1, Rc and Rb2 are consistently higher in Huanren than in the other two origins. They can serve as candidate markers for Huanren.

For Tonghua vs. Ji’an: These two origins are difficult to distinguish by ginsenosides at 15 years. However, at 20 years, Tonghua samples contain significantly higher Re than Ji’an. Thus, Re acts as an age-specific marker that is informative only at 20 years.

Implications for practical authentication:

For volatile-based authentication, a full multivariate model is necessary; it is not possible to rely on one or two individual compounds.

For ginsenoside-based authentication, one can first examine Rf, Rg1, Rc and Rb2 to identify Huanren; for 20-year samples, Re can further discriminate Tonghua from Ji’an. This approach is more flexible and does not always require complex modeling.

In short, volatile information is “distributed” and must be interpreted as a whole; ginsenoside information is “locally readable” and allows tiered discrimination using a few key compounds. The two approaches have complementary strengths and weaknesses.

### 4.4. A Practical Two-Step Authentication Strategy

The complementary behavior of the volatile and ginsenoside fractions, combined with the hierarchical marker structure identified in the latter, suggests a practical two-tier authentication workflow.

At the first tier, the ginsenoside profile provides a rapid, interpretable geographical screening. These candidate markers (Rf, Rg1, Rc, and Rb2) could serve as a chemical barcode for Huanren origin that is largely independent of growth year, pending validation in larger and more diverse sample sets. For samples where Ji’an and Tonghua must be distinguished—a task that becomes necessary primarily for 20-year-old ginseng—Re provides a single-compound diagnostic, with elevated Re indicating Tonghua origin. This ginsenoside-based screening can be implemented with targeted HPLC analysis and does not require complex multivariate modeling.

At the second tier, the volatile fingerprint provides high-resolution, age-specific confirmation within the PLS-DA latent variable space. For samples that fall near decision boundaries or for applications requiring the highest confidence, the full volatile fingerprint analyzed by the corresponding age-matched PLS-DA model delivers the definitive origin assignment.

Critically, both tiers independently require growth-year information. The ginsenoside-based tier requires it because Re only discriminates Ji’an from Tonghua at 20 years; the volatile-based tier requires it because age-matched models achieve 100% separation while mixed-age models would suffer from reduced specificity. The two-step workflow—growth year determination by morphological inspection or chemical prediction, followed by age-matched origin assignment—operationalizes this requirement. This integrated strategy converts the biological reality of year-dependent metabolic variation from an analytical obstacle into a structured component of the authentication framework.

### 4.5. Limitations and Future Directions

Several limitations of this study should be acknowledged. First, the sample size, although carefully designed with three replicates per group (n = 18), was constrained by the scarcity and high cost of authentic wild ginseng material. The use of pooled samples—each consisting of five individual roots homogenized into a single analytical sample—provided representative group-level chemical profiles but precluded the assessment of biological variability within each origin × year combination. A further limitation is that all samples were collected in a single year (August 2025). Wild ginseng chemical profiles are known to be influenced by inter-annual climatic variations (e.g., temperature, precipitation). Therefore, the robustness of the identified markers and the specific PLS-DA models across multiple harvest years cannot be guaranteed. Future studies should incorporate samples collected over several consecutive years to assess the temporal stability of the chemical fingerprints and the discriminant models.

Second, the geographical coverage was limited to three origins. The selection of sampling areas was based on climatic conditions, with regions from Jilin and Liaoning provinces—China’s most important ginseng production areas—with similar climates chosen to investigate the impact of minimal environmental differences on geographical traceability. However, the sampling still did not cover the entire range of Northeast China’s ginseng-producing regions, failing to comprehensively reflect the overall quality variations in wild ginseng across different production areas nationwide. Future studies should expand the sampling scope to include samples from regions with diverse latitudes, altitudes, soil types, and longer aging periods (e.g., 10 years, 25 years), thereby further validating the findings of this study and refining the analysis of how microenvironmental factors influence the chemical characteristics of WG.

Third, only seven major ginsenosides were quantified by HPLC; untargeted metabolomics approaches (e.g., UPLC-QTOF-MS) could complement the targeted analysis in future studies.

Fourth, while the parallel analysis of volatile and ginsenoside datasets effectively demonstrated the convergent necessity of age stratification, several analytical limitations should be acknowledged. The two datasets were integrated only at the interpretive level, without formal data fusion algorithms (e.g., mid-level fusion combining latent variables from both platforms), which could potentially enhance discrimination accuracy as shown in prior food authentication studies [37,38]. Moreover, the observed origin × year interaction in the volatile dataset remains largely descriptive, as it was not subjected to inferential statistical confirmation (e.g., via permutation-based tests of interaction effects). Most importantly, although our PLS-DA results strongly suggest that discriminatory power may reside in the multivariate relationships among compounds, the present study did not perform formal analyses—such as correlation network stability tests, Box’s M-test, or multi-block covariance structure analysis—to directly validate the existence of a year-invariant covariance structure. Consequently, the interpretation that geographical origin information is encoded in the covariance structure should be regarded as a hypothesis requiring direct testing in future studies, ideally with independent sampling and sufficient biological replication across multiple growth years and origins.

## 5. Conclusions

This study indicates that both volatile (HS-GC-IMS) and non-volatile (HPLC ginsenoside) chemical fingerprints of WG carry robust geographical origin information, but that this information is clearly influenced by growth year.

The key findings are as follows:

(1) Volatile fingerprinting by HS-GC-IMS coupled with age-stratified PLS-DA achieved complete origin separation of Huanren, Tonghua, and Ji’an WG. Ten shared VIP markers were identified, yet none exhibited a consistent origin ranking across growth years, yet none exhibited a consistent origin ranking across growth years, indicating that multivariate patterns, rather than any single invariant marker, are required for reliable origin discrimination.
(2) Ginsenoside profiling by HPLC provided a complementary, pharmacopeia-aligned origin signature with a hierarchical marker system. Rf, Rg1, Rc, and Rb2 were identified as the most robust candidate universal origin markers, consistently associated with Huanren across growth years. Rb1 and Rd were additional candidate universal markers with moderate age-dependency, while Re emerged as a 20-year-specific marker uniquely capable of discriminating Ji’an from Tonghua. This hierarchical structure underscores the dimension-specific roles of individual ginsenosides in geographical differentiation.
(3) The origin × growth year interaction is pervasive across both chemical fractions, indicating that age-matched, stratified chemometric models are a necessity for reliable WG origin authentication. A practical two-step workflow—growth year determination followed by age-specific origin assignment—is proposed.

These findings provide a scientific foundation for the geographical traceability of wild ginseng. While further validation across additional harvest years and regions is needed, the proposed age-stratified strategy offers a robust framework for authentication.

## Figures and Tables

**Table 1:** Volatile compounds identified in wild ginseng by HC-GC-IMS.

Category	Compound	CAS#	Formula	MW	RI	Rt/s	Dt/ms	Odor Description
Terpenes	Nerolidol	C7212444	C_15_H_26_O	222.4	1541.1	2337.605	1.49178	flower, green, waxy, citrus aroma, woody flavor
	(E, E)-α-Farnesene	C502614	C_15_H_24_	204.4	1518.8	2217.096	1.43251	citrus herbal lavender bergamot myrrh neroli green
	d-Longifolene	C475207	C_15_H_24_	204.4	1407.4	1702.666	1.44243	sweet woody rose medical fir needle
	Iso-Longifolene	C1135666	C_15_H_24_	204.4	1371.9	1565.467	1.44243	woody
	(+)-Limonene M	C138863	C_10_H_16_	136.2	1033.9	702.724	1.21679	lemon, sweet, orange, pine oil
	(+)-Limonene D	C138863	C_10_H_16_	136.2	1034.1	703.113	1.29548	lemon, sweet, orange, pine oil
	Camphene	C79925	C_10_H_16_	136.2	954.5	550.744	1.209	woody, camphor
	(R)-α-pinene	C7785708	C_10_H_16_	136.2	937.1	516.454	1.21259	Terpenic, Mint, Pine
	β-Pinene M	C127913	C_10_H_16_	136.2	983.7	613.341	1.21737	resin, green
	β-Pinene D	C127913	C_10_H_16_	136.2	984.5	615.313	1.29282	resin, green
	β-Pinene P	C127913	C_10_H_16_	136.2	983.8	613.67	1.72327	resin, green
	β-ionone	C14901076	C_13_H_20_O	192.3	1499	2115.677	1.47531	rose, floral, iris, fruity, woody
	(-)-Carvone	C99490	C_10_H_14_O	150.2	1246.6	1163.382	1.31536	spearmint
	Carveol	C99489	C_10_H_16_O	152.2	1217.7	1086.292	1.29358	fresh, spearmint, caraway
	Linalool	C78706	C_10_H_18_O	154.3	1100.2	822.177	1.25066	citrus, rose, woody, blueberry
Ketones	3-Octanone	C106683	C_8_H_16_O	128.2	998.1	645.59	1.70914	moldy, ketone, green, waxy, vegetable, mushroom, fruity
	3-Heptanone M	C106354	C_7_H_14_O	114.2	890.2	434.486	1.23525	Fruity, Grass, Oil
	3-Heptanone D	C106354	C_7_H_14_O	114.2	889	432.685	1.58984	Fruity, Grass, Oil
	2-Hexanone	C591786	C_6_H_12_O	100.2	804.6	323.427	1.19587	fruity, fungal, meaty, buttery
	2-butanone 3-hydroxy M	C513860	C_4_H_8_O_2_	88.1	727.6	246.218	1.06235	butter, cream
	2-butanone 3-hydroxy D	C513860	C_4_H_8_O_2_	88.1	719.2	238.956	1.33411	butter, cream
	2-Pentanone	C107879	C_5_H_10_O	86.1	689.2	214.626	1.11425	acetone, fresh, sweet fruity, wine
	2,3-Pentanedione	C600146	C_5_H_8_O_2_	100.1	714.5	235.015	1.22865	sweet, cream, caramel, nuts, cheese
	2,3-butanedione	C431038	C_4_H_6_O_2_	86.1	589.3	166.606	1.17706	butter, popcorn, sweet taste, sour rice
	2-Butanone D	C78933	C_4_H_8_O	72.1	596.9	169.852	1.24222	fruity, camphor
	2-Butanone M	C78933	C_4_H_8_O	72.1	596.9	169.852	1.05683	fruity, camphor
	3-Pentanone	C96220	C_5_H_10_O	86.1	694.2	218.547	1.35976	ethereal
	2-pentanone, 3-methyl	C565617	C_6_H_12_O	100.2	747.8	264.748	1.46842	mint, honey
	4-methyl-3-Penten-2-one	C141797	C_6_H_10_O	98.1	797.3	315.419	1.43959	spice, earth, green
	1 -Hydroxy-2-propanone	C116096	C_3_H_6_O_2_	74.1	685.5	212.523	1.04191	pungent, caramel, fresh
Aldehydes	(*E*)-2-Octenal	C2548870	C_8_H_14_O	126.2	1064.1	754.849	1.33015	fresh cucumber, fatty, green herbal, banana, green leaf
	(*E*)-Hept-2-enal	C18829555	C_7_H_12_O	112.2	964.5	571.404	1.65754	spicy, green vegetables, fresh, fatty
	(*E*)-2-Hexenal M	C6728263	C_6_H_10_O	98.1	854.2	383.795	1.17996	green, banana, fat
	(*E*)-2-Hexenal D	C6728263	C_6_H_10_O	98.1	852.8	381.993	1.51532	green, banana, fat
	3-Methyl-2-butenal D	C107868	C_5_H_8_O	84.1	784.8	302.125	1.35719	fruity
	3-Methyl-2-butenal M	C107868	C_5_H_8_O	84.1	783.9	301.197	1.09391	fruity
	*Trans*-2-pentenal M	C1576870	C_5_H_8_O	84.1	754.6	271.19	1.1033	potato, peas
	*Trans*-2-pentenal D	C1576870	C_5_H_8_O	84.1	753	269.656	1.36007	potato, peas
	Heptanal M	C111717	C_7_H_14_O	114.2	906.1	460.475	1.33381	fresh, aldehyde, fatty, green herbs, wine, fruity
	Heptanal D	C111717	C_7_H_14_O	114.2	905	458.674	1.68961	fresh, aldehyde, fatty, green herbs, wine, fruity
	Hexanal M	C66251	C_6_H_12_O	100.2	802.9	321.554	1.26234	fresh, green, fat, fruity
	Hexanal D	C66251	C_6_H_12_O	100.2	797.3	315.419	1.56223	fresh, green, fat, fruity
	Butanal	C123728	C_4_H_8_O	72.1	606.5	174.038	1.28922	pungent, fruity, green leaf
	Propanal	C123386	C_3_H_6_O	58.1	493.6	130.788	1.05642	pungent, green grassy
	n-Pentanal M	C110623	C_5_H_10_O	86.1	699.2	222.482	1.19633	green grassy, faint banana, pungent
	n-Pentanal D	C110623	C_5_H_10_O	86.1	698.9	222.224	1.4229	green grassy, faint banana, pungent
	2-Methylpropanal	C78842	C_4_H_8_O	72.1	571.4	159.218	1.28734	banana, melon, slightly nutty
	2-Phenylacetaldehyde	C122781	C_8_H_8_O	120.2	1048.5	727.397	1.25277	hyacinth, sweet fruity, almond, cherry, clover honey, cocoa
Alcohols	1-Hexanol M	C111273	C_6_H_14_O	102.2	874.3	411.328	1.3242	fresh, fruity, wine, sweet, green
	1-Hexanol D	C111273	C_6_H_14_O	102.2	873.4	410.041	1.64033	fresh, fruity, wine, sweet, green
	1-Pentanol M	C71410	C_5_H_12_O	88.1	765.7	282.183	1.25372	balsamic
	1-Pentanol D	C71410	C_5_H_12_O	88.1	766.2	282.695	1.51337	balsamic
	1-Butanol M	C71363	C_4_H_10_O	74.1	664.8	201.668	1.18155	wine
	1-Butanol D	C71363	C_4_H_10_O	74.1	661.8	200.144	1.37446	wine
	1-Propanol	C71238	C_3_H_8_O	60.1	533.1	144.55	1.11957	alcohol, pungent
	3-Methyl butanol M	C123513	C_5_H_12_O	88.1	734.9	252.73	1.23831	whiskey, banana, fruity
	3-Methyl butanol D	C123513	C_5_H_12_O	88.1	735.1	252.98	1.49052	whiskey, banana, fruity
	1-Propanol, 2-methyl	C78831	C_4_H_10_O	74.1	630.3	184.825	1.17479	fresh, alcoholic, leather
	1-Penten-3-ol	C616251	C_5_H_10_O	86.1	686.7	213.184	0.93418	ethereal, green, tropical fruity
Esters	Acetic acid, hexyl ester	C142927	C_8_H_16_O_2_	144.2	1012.4	667.852	1.40698	fruity, green, apple, banana, sweet
Esters	Hexanoic acid methyl ester	C106707	C_7_H_14_O_2_	130.2	937.2	516.526	1.27887	pineapple, apricot, fruity
	Isobutyl propanoate	C540421	C_7_H_14_O_2_	130.2	863.6	396.403	1.2641	rum, pineapple
	Ethyl 2-methylbutanoate	C7452791	C_7_H_14_O_2_	130.2	857.5	388.169	1.23645	apple
	Methyl 2-methyl butyrate	C868575	C_6_H_12_O_2_	116.2	777.6	294.455	1.18953	apple
	Ac. acetic ethyl ester M	C141786	C_4_H_8_O_2_	88.1	619.7	179.945	1.09967	fresh, fruity, sweet, grassy
	Ac. acetic ethyl ester D	C141786	C_4_H_8_O_2_	88.1	618.5	179.373	1.33521	fresh, fruity, sweet, grassy
	n-Propyl acetate	C109604	C_5_H_10_O_2_	102.1	713.7	234.268	1.16076	fruity, pear
	(*E*)-3-Hexenyl acetate	C3681718	C_8_H_14_O_2_	142.2	1012	667.231	1.82102	fresh green grassy, sweet, fruity, banana
	n-Butyl lactate	C34451199	C_7_H_14_O_3_	146.2	1019.8	679.633	1.26842	sweet, fruity
	2-Furanmethanol acetate	C623176	C_7_H_8_O_3_	140.1	992.9	634.545	1.42368	sweet, banana
Pyrazines	2,3,5-Trimethylpyrazine	C14667551	C_7_H_10_N_2_	122.2	1003.7	654.171	1.16189	roasted potato, peanut, cocoa, chocolate
	2-Ethyl-6-methylpyrazine	C13925036	C_7_H_10_N_2_	122.2	994.8	639.097	1.17084	nutty, roast, sweet
	2,5-Dimethylpyrazine	C123320	C_6_H_8_N_2_	108.1	896.5	444.522	1.10784	nutty, peanut, moldy, earthy, potato, fatty, cocoa powder
organic acid	3-Methyl valeric acid	C105431	C_6_H_12_O_2_	116.2	945.5	532.616	1.59647	sour, herbal, slight green
	Acetic acid	C64197	C_2_H_4_O_2_	60.1	633.7	186.424	1.04583	spicy
	3-Methylbutanoic acid	C503742	C_5_H_10_O_2_	102.1	875.2	412.65	1.48565	sour, foot sweat, cheese
Others	1,2-Dimethoxyethane	C110714	C_4_H_10_O_2_	90.1	655.2	196.816	1.30428	ether
	Allyl propyl sulfide	C27817670	C_6_H_12_S	116.2	873.8	410.567	1.39721	garlic, onion
	2-Pentylfuran	C3777693	C_9_H_14_O	138.2	999.1	647.111	1.25551	bean, fruity, earthy, green, vegetable
	4,5-Dihydro-2-methyl-3(2 H)thiophenone	C13679851	C_5_H_8_OS	116.2	998.4	646.054	1.21561	cabbage, onion, must
	2-Phenyl-1,3-dioxolane-4-methanol M	C1708390	C_10_H_12_O_3_	180.2	967.8	578.487	1.14923	sweet berries, bitter almonds
	2-Phenyl-1.3-dioxolane-4-methanol D	C1708390	C_10_H_12_O_3_	180.2	967.3	577.271	1.46389	sweet berries, bitter almonds
	2,2,4,6,6-Pentamethylheptane	C13475826	C_12_H_26_	170.3	990.3	628.653	1.15566	tasteless
	2,4-Dimethylheptane	C2213232	C_9_H_20_	128.3	824.5	346.427	1.20149	gasoline
	1-Heptene	C592767	C_7_H14	98.2	687.3	213.482	1.07836	gasoline

Note: The suffixes M, D, or P denote the monomer, dimer, and polymer forms of the same compound. Odor descriptions were primarily obtained from the database of https://www.flavornet.org (accessed on 15 December 2025).

**Table 2:** Variation in the content of volatile components in wild ginseng samples under the interaction of origin and growth years.

Category	Compounds	15 Years WG, Content, Mean ± SD (mg/kg)			20 Years WG, Content, Mean ± SD (mg/kg)		
		Huanren County	Tonghua County	Jian City	Huanren County	Tonghua County	Jian City
Terpenes	Nerolidol	1.76 ± 0.006 ^a^	1.64 ± 0.093 ^ab^	1.51 ± 0.107 ^b^	1.63 ± 0.025 ^a^	1.65 ± 0.083 ^a^	1.75 ± 0.024 ^a^
Terpenes	(E,E)-α-Farnesene	1.73 ± 0.035 ^a^	1.39 ± 0.03 ^b^	1.38 ± 0.159 ^b^	1.64 ± 0.026 ^a^	1.4 ± 0.04 ^b^	1.47 ± 0.022 ^b^
Terpenes	d-Longifolene	2.81 ± 0.04 ^a^	2.34 ± 0.081 ^b^	2.18 ± 0.29 ^b^	2.94 ± 0.084 ^a^	2.26 ± 0.09 ^b^	2.26 ± 0.034 ^b^
Terpenes	(+)-Limonene D	0.11 ± 0.006 ^a^	0.13 ± 0 ^b^	0.15 ± 0.006 ^c^	0.11 ± 0.005 ^ab^	0.12 ± 0 ^a^	0.11 ± 0 ^b^
Terpenes	Camphene	0.54 ± 0 ^b^	0.52 ± 0.02 ^b^	0.58 ± 0.01 ^a^	1.04 ± 0.009 ^a^	0.39 ± 0.008 ^b^	0.42 ± 0.014 ^b^
Terpenes	(R)-α-pinene	0.58 ± 0 ^b^	0.6 ± 0.021 ^ab^	0.62 ± 0.006 ^a^	0.72 ± 0.005 ^a^	0.53 ± 0.005 ^c^	0.57 ± 0.005 ^b^
Terpenes	β-Pinene P	0.62 ± 0.015 ^a^	0.65 ± 0.031 ^a^	0.59 ± 0.084 ^a^	0.68 ± 0.029 ^a^	0.64 ± 0.012 ^ab^	0.61 ± 0.008 ^b^
Ketones	β-ionone	2.08 ± 0.038 ^a^	1.51 ± 0.06 ^b^	1.38 ± 0.101 ^b^	1.65 ± 0.024 ^a^	1.46 ± 0.067 ^b^	1.37 ± 0.059 ^c^
Ketones	(-)-Carvone	0.77 ± 0.015 ^a^	0.43 ± 0.02 ^b^	0.42 ± 0.081 ^b^	0.58 ± 0.009 ^a^	0.4 ± 0.031 ^b^	0.42 ± 0.017 ^b^
Ketones	3-Octanone	0.13 ± 0.006 ^a^	0.11 ± 0.006 ^a^	0.09 ± 0.006 ^b^	0.15 ± 0.005 ^a^	0.1 ± 0.005 ^b^	0.12 ± 0.008 ^b^
Ketones	2-Pentanone	0.23 ± 0.006 ^a^	0.28 ± 0.015 ^b^	0.26 ± 0.015 ^b^	0.22 ± 0.005 ^c^	0.31 ± 0.005 ^b^	0.89 ± 0.016 ^a^
Ketones	5-Methyl-3-heptanone	2.7 ± 0.04 ^a^	2.17 ± 0.05 ^b^	2.52 ± 0.103 ^c^	2.8 ± 0.012 ^a^	2.38 ± 0.123 ^b^	1.99 ± 0.038 ^c^
Ketones	3-Heptanone D	0.65 ± 0.02 ^b^	0.75 ± 0.021 ^a^	0.76 ± 0.012 ^a^	0.76 ± 0.014 ^a^	0.76 ± 0.014 ^a^	0.59 ± 0.029 ^b^
Ketones	2-Hexanone	0.06 ± 0 ^c^	0.2 ± 0.023 ^b^	0.43 ± 0.006 ^a^	0.12 ± 0.005 ^b^	0.25 ± 0 ^a^	0.25 ± 0.005 ^a^
Ketones	2-Butanone 3-hydroxy D	1.72 ± 0.023 ^c^	6.81 ± 0.229 ^a^	3.96 ± 0.055 ^b^	2.21 ± 0.005 ^c^	3.65 ± 0.062 ^b^	4.75 ± 0.07 ^a^
Ketones	1-Hydroxy-2-propanone	0.63 ± 0.017 ^c^	0.96 ± 0.012 ^b^	1.04 ± 0.038 ^a^	1.02 ± 0.008 ^b^	0.89 ± 0.009 ^c^	1.27 ± 0.057 ^a^
Ketones	2,3-Butandione	0.6 ± 0.029 ^a^	0.73 ± 0.051 ^a^	0.62 ± 0.098 ^a^	0.71 ± 0.033 ^b^	0.66 ± 0.054 ^b^	0.98 ± 0.065 ^a^
Ketones	2-Butanone D	2.13 ± 0.102 ^a^	1.44 ± 0.159 ^b^	0.9 ± 0.24 ^c^	1.73 ± 0.069 ^a^	1.27 ± 0.107 ^b^	0.93 ± 0.028 ^c^
Ketones	3-Penten-2-one, 4-methyl-	0.81 ± 0.012 ^b^	0.85 ± 0.015 ^a^	0.58 ± 0.015 ^c^	0.85 ± 0.016 ^a^	0.68 ± 0.017 ^b^	0.62 ± 0.005 ^c^
Ketones	2,3-Pentanedione	0.35 ± 0.006 ^a^	0.62 ± 0.006 ^b^	0.52 ± 0.053 ^c^	0.49 ± 0.005 ^b^	0.47 ± 0 ^b^	0.93 ± 0.036 ^a^
Ketones	2-Pentanone, 3-methyl	0.01 ± 0.006 ^b^	0.02 ± 0.006 ^b^	0.04 ± 0 ^a^	0.02 ± 0 ^c^	0.04 ± 0 ^a^	0.03 ± 0 ^b^
Aldehydes	(*E*)-2-Octenal	0.33 ± 0.012 ^a^	0.2 ± 0.012 ^b^	0.09 ± 0.023 ^c^	0.23 ± 0 ^a^	0.12 ± 0.008 ^b^	0.1 ± 0.005 ^c^
Aldehydes	(*E*)-Hept-2-enal	0.49 ± 0.067 ^a^	0.19 ± 0.081 ^b^	0.16 ± 0.116 ^b^	0.37 ± 0.017 ^a^	0.13 ± 0.042 ^b^	0.1 ± 0.019 ^b^
Aldehydes	Heptanal D	4.13 ± 0.238 ^a^	1.91 ± 0.336 ^b^	1.03 ± 0.428 ^c^	3.73 ± 0.117 ^a^	1.3 ± 0.191 ^b^	1.73 ± 0.222 ^c^
Aldehydes	(*E*)-2-Hexenal D	1.34 ± 0.025 ^a^	0.36 ± 0.012 ^b^	0.3 ± 0.017 ^c^	1.03 ± 0.005 ^a^	0.22 ± 0.012 ^c^	0.28 ± 0.012 ^b^
Aldehydes	Hexanal D	9.31 ± 0.079 ^a^	5.82 ± 0.067 ^b^	2.91 ± 0.226 ^c^	7.9 ± 0.05 ^a^	4.39 ± 0.008 ^b^	2.93 ± 0.057 ^c^
Aldehydes	*Trans*-2-pentenal D	0.16 ± 0.006 ^a^	0.14 ± 0.006 ^b^	0.04 ± 0 ^c^	0.14 ± 0 ^a^	0.05 ± 0.005 ^b^	0.07 ± 0.005 ^c^
Aldehydes	n-pentanal D	2.56 ± 0.025 ^a^	0.87 ± 0.052 ^b^	0.4 ± 0.119 ^c^	1.43 ± 0.037 ^a^	0.7 ± 0.037 ^b^	0.24 ± 0.019 ^c^
Aldehydes	Butanal	0.92 ± 0.017 ^a^	0.72 ± 0.038 ^b^	0.51 ± 0.08 ^c^	0.99 ± 0.039 ^a^	0.68 ± 0.012 ^b^	0.4 ± 0.014 ^c^
Aldehydes	Propanal	2.7 ± 0.058 ^b^	2.3 ± 0.167 ^c^	3.43 ± 0.01 ^a^	3.53 ± 0.171 ^a^	3.11 ± 0.058 ^b^	1.1 ± 0.026 ^c^
Aldehydes	3-Methyl-2-butenal-D	1.47 ± 0.015 ^b^	1.88 ± 0 ^a^	1.06 ± 0.032 ^c^	1.67 ± 0.021 ^b^	1.37 ± 0.017 ^c^	2.01 ± 0.025 ^a^
Aldehydes	2-Methyl propanal	0.14 ± 0.021 ^a^	0.11 ± 0.012 ^a^	0.13 ± 0.025 ^a^	0.08 ± 0.009 ^b^	0.11 ± 0.008 ^a^	0.06 ± 0.008 ^b^
Esters	n-Butyl lactate	0.16 ± 0.01 ^b^	0.21 ± 0.01 ^a^	0.22 ± 0.015 ^a^	0.16 ± 0.012 ^b^	0.2 ± 0.005 ^a^	0.2 ± 0.008 ^a^
Esters	Acetic acid, hexyl ester	2.93 ± 0.044 ^a^	1.97 ± 0.071 ^b^	1.64 ± 0.26 ^b^	2.84 ± 0.017 ^a^	1.78 ± 0.086 ^b^	1.55 ± 0.065 ^c^
Esters	(*E*)-3-Hexenyl acetate	2.6 ± 0.137 ^a^	0.9 ± 0.08 ^b^	0.48 ± 0.205 ^c^	2.3 ± 0.029 ^a^	0.58 ± 0.067 ^b^	0.54 ± 0.071 ^b^
Esters	Ethyl 2-methyl butanoate	0.19 ± 0 ^a^	0.07 ± 0.006 ^b^	0.08 ± 0.006 ^b^	0.14 ± 0.005 ^a^	0.06 ± 0.005 ^b^	0.06 ± 0.005 ^b^
Esters	Isobutyl propanoate	0.15 ± 0 ^a^	0.11 ± 0.006 ^b^	0.07 ± 0 ^c^	0.1 ± 0.005 ^a^	0.1 ± 0.005 ^a^	0.06 ± 0.005 ^b^
Esters	Methyl 2-methyl butyrate	1 ± 0.021 ^b^	0.75 ± 0.038 ^c^	1.42 ± 0.031 ^a^	1.7 ± 0.005 ^a^	1.2 ± 0.008 ^b^	1.53 ± 0.019 ^c^
Esters	Ac. acetic ethyl ester D	2.21 ± 0.105 ^c^	3.01 ± 0.059 ^b^	3.68 ± 0.14 ^a^	3.99 ± 0.033 ^b^	3.49 ± 0.057 ^c^	8.27 ± 0.08 ^a^
Esters	n-Propyl acetate	0.17 ± 0.006 ^a^	0.11 ± 0.012 ^b^	0.13 ± 0.015 ^b^	0.1 ± 0.005 ^c^	0.18 ± 0.005 ^a^	0.15 ± 0.008 ^b^
Esters	Hexanoic acid methyl ester	1.27 ± 0.015 ^a^	0.99 ± 0.03 ^b^	1.22 ± 0.015 ^a^	1.23 ± 0.017 ^a^	1.18 ± 0.017 ^b^	1 ± 0.014 ^c^
Esters	2-Furanmethanol acetate	0.05 ± 0 ^a^	0.04 ± 0.01 ^a^	0.03 ± 0.006 ^a^	0.04 ± 0 ^a^	0.04 ± 0.005 ^a^	0.03 ± 0.005 ^a^
Alcohols	Carveol	0.29 ± 0.017 ^a^	0.15 ± 0.006 ^b^	0.15 ± 0.02 ^b^	0.14 ± 0.009 ^a^	0.14 ± 0.009 ^a^	0.13 ± 0.005 ^a^
Alcohols	1-Hexanol D	0.94 ± 0.006 ^a^	0.9 ± 0.02 ^a^	0.93 ± 0.021 ^a^	1.19 ± 0.008 ^a^	0.99 ± 0.017 ^b^	0.7 ± 0.025 ^c^
Alcohols	Linalool	0.42 ± 0.015 ^a^	0.17 ± 0.01 ^b^	0.14 ± 0 ^c^	0.46 ± 0.005 ^a^	0.15 ± 0.009 ^b^	0.26 ± 0 ^c^
Alcohols	1-Pentanol D	0.35 ± 0.006 ^a^	0.3 ± 0.006 ^b^	0.12 ± 0.01 ^c^	0.26 ± 0.009 ^a^	0.2 ± 0.009 ^b^	0.12 ± 0.005 ^c^
Alcohols	3-Methyl butanol D	0.03 ± 0 ^c^	0.28 ± 0.01 ^b^	0.43 ± 0.012 ^a^	0.07 ± 0.005 ^b^	0.37 ± 0.005 ^a^	0.36 ± 0.008 ^a^
Alcohols	1-Butanol D	5.03 ± 0.035 ^a^	2.19 ± 0.086 ^b^	2.18 ± 0.095 ^b^	3.18 ± 0.021 ^a^	2.55 ± 0.012 ^b^	1.46 ± 0.042 ^c^
Alcohols	1-Propanol, 2-methyl	0.13 ± 0.006 ^b^	0.13 ± 0 ^b^	0.19 ± 0.006 ^a^	0.12 ± 0.005 ^b^	0.22 ± 0.009 ^a^	0.11 ± 0.005 ^b^
Alcohols	1-Propanol	14.07 ± 0.261 ^a^	8 ± 0.256 ^b^	8.43 ± 0.303 ^b^	10.51 ± 0.095 ^a^	8.74 ± 0.074 ^b^	6.59 ± 0.109 ^c^
Alcohols	1-Penten-3-ol	0.36 ± 0.006 ^a^	0.24 ± 0.006 ^b^	0.11 ± 0.015 ^c^	0.33 ± 0.005 ^a^	0.17 ± 0.005 ^b^	0.07 ± 0 ^c^
Pyrazines	2, 3, 5-Trimethyl pyrazine	0.59 ± 0.031 ^a^	0.52 ± 0.068 ^a^	0.23 ± 0.058 ^b^	0.65 ± 0.012 ^a^	0.38 ± 0.033 ^b^	0.45 ± 0.04 ^b^
Pyrazines	2,5-Dimethyl pyrazine	0.38 ± 0.026 ^b^	0.56 ± 0.074 ^a^	0.23 ± 0.04 ^c^	0.46 ± 0.016 ^a^	0.4 ± 0.045 ^a^	0.5 ± 0.039 ^a^
Pyrazines	2-Ethyl-6-methyl pyrazine	0.42 ± 0.015 ^a^	0.16 ± 0.015 ^b^	0.16 ± 0.029 ^b^	0.29 ± 0.009 ^a^	0.14 ± 0.005 ^c^	0.16 ± 0.005 ^b^
Organic acids	3-Methyl valeric acid	0.24 ± 0 ^b^	0.36 ± 0.012 ^a^	0.36 ± 0 ^a^	0.24 ± 0.009 ^c^	0.34 ± 0.005 ^b^	0.38 ± 0.005 ^a^
Organic acids	Acetic acid	1.39 ± 0.035 ^b^	1.87 ± 0.062 ^a^	1.82 ± 0.015 ^a^	2.06 ± 0.022 ^a^	1.67 ± 0.031 ^c^	1.8 ± 0.034 ^b^
Organic acids	3-Methylbutanoic acid	0.13 ± 0.006 ^b^	0.14 ± 0 ^a^	0.13 ± 0.006 ^b^	0.17 ± 0 ^a^	0.14 ± 0.005 ^b^	0.17 ± 0.005 ^a^
others	1-Heptene	0.26 ± 0.006 ^a^	0.28 ± 0.006 ^a^	0.26 ± 0.021 ^a^	0.27 ± 0.005 ^a^	0.27 ± 0.009 ^a^	0.26 ± 0.009 ^a^
others	2-Pentyl furan	0.24 ± 0.015 ^a^	0.15 ± 0.017 ^b^	0.09 ± 0.006 ^c^	0.21 ± 0.005 ^a^	0.11 ± 0.008 ^b^	0.12 ± 0.005 ^b^
others	1,2-Dimethoxyethane	1.47 ± 0.036 ^c^	2.58 ± 0.04 ^b^	1.74 ± 0.006 ^a^	1.83 ± 0.005 ^c^	2.09 ± 0.022 ^b^	2.36 ± 0.029 ^a^
others	Hexanenitrile	0.05 ± 0 ^a^	0.05 ± 0.006 ^ab^	0.04 ± 0.006 ^b^	0.06 ± 0.005 ^a^	0.04 ± 0 ^c^	0.05 ± 0.005 ^b^
others	Diethyl disulfide	0.22 ± 0.01 ^a^	0.21 ± 0.006 ^a^	0.21 ± 0.006 ^a^	0.21 ± 0.005 a	0.19 ± 0.005 ^a^	0.13 ± 0.005 ^b^
others	2,4-Dimethyl heptane	0.1 ± 0 ^c^	0.14 ± 0.012 ^b^	0.17 ± 0.006 ^a^	0.19 ± 0.005 ^b^	0.16 ± 0.005 ^a^	0.21 ± 0.005 ^c^
others	4,5-Dihydro-2-methyl-3(2 H)thiophenone	0.35 ± 0.006 ^b^	0.45 ± 0.017 ^a^	0.45 ± 0.015 ^a^	0.5 ± 0.005 ^b^	0.5 ± 0 ^b^	0.6 ± 0.008 ^a^
others	2,2,4,6,6-Pentamethyl heptane	0.19 ± 0.006 ^a^	0.16 ± 0.01 ^a^	0.08 ± 0.032 ^b^	0.17 ± 0.008 ^a^	0.1 ± 0.014 b	0.06 ± 0.008 ^c^
others	n-Propyl benzene	0.67 ± 0.032 ^a^	0.4 ± 0.081 ^b^	0.36 ± 0.159 ^b^	0.55 ± 0.009 ^a^	0.32 ± 0.068 ^b^	0.27 ± 0.04 ^b^

Note: The superscripted letters a–c are for the different subgroups.

## Data Availability

The raw data supporting the conclusions of this article will be made available by the authors on request.

## Supplementary Materials

The following supporting information can be downloaded at: https://www.mdpi.com/article/10.3390/foods15132310/s1, Figure S1: Permutation test validation of ginsenosides confirming the robustness and non-overfitting of PLS-DA models for the geographical origin of WG across different growth years; Table S1: 15 Years of Volatile Compounds Content; Table S2: 20 Years of Volatile Compounds Content; Table S3: 15 Years of ginsenosides Content; Table S4: 15 Years of ginsenosides Content.

## Abbreviations

The following abbreviations are used in this manuscript:

WG	wild ginseng
HS-GC-IMS	headspace gas chromatography-ion mobility spectrometry
PLS-DA	partial least squares-discriminant analysis
HPLC	high-performance liquid chromatography
ANOVA	One-way analysis of variance
VIP	variable importance in projection
