# Supplementary material for: Geographical Origin Authentication of Wild Ginseng by Volatile and Non-Volatile Fingerprinting Across Growth Years
Source: Foods. 2026 Jun 29;15(13):2310. doi: 10.3390/foods15132310 (PMC13361996; doi:10.3390/foods15132310)
Supplement: Supplementary file 1 [file foods-15-02310-s001.zip › Dataset.pdf]

Table S1. 15 Years of Volatile Compounds Content

Compounds	gs10-1	gs10-2	gs10-3	gs18-1	gs18-2	gs18-3	gs19-1	gs19-2	gs19-3
Nerolidol	1.77	1.76	1.76	1.75	1.58	1.60	1.60	1.53	1.39
(E,E)-alpha-Farnesene	1.73	1.70	1.77	1.42	1.39	1.36	1.50	1.44	1.20
β-ionone	2.10	2.11	2.04	1.57	1.50	1.45	1.37	1.49	1.29
d-Longifolene	2.77	2.80	2.85	2.40	2.38	2.25	2.38	2.32	1.85
(-)-Carvone	0.77	0.75	0.78	0.45	0.43	0.41	0.48	0.46	0.33
Carveol	0.28	0.28	0.31	0.15	0.14	0.15	0.17	0.15	0.13
Linalool	0.42	0.41	0.44	0.17	0.16	0.18	0.14	0.14	0.14
(E)-2-Octenal	0.32	0.34	0.32	0.21	0.19	0.21	0.08	0.08	0.12
(+)-Limonene D	0.11	0.10	0.11	0.13	0.13	0.13	0.15	0.15	0.14
n-Butyl lactate	0.17	0.15	0.16	0.22	0.21	0.20	0.23	0.22	0.20
Acetic acid, hexyl ester	2.91	2.98	2.90	1.98	1.89	2.03	1.48	1.50	1.94
(Z)-3-Hexenyl acetate	2.57	2.75	2.48	0.89	0.82	0.98	0.36	0.37	0.72
2, 3, 5-Trimethylpyrazine	0.58	0.62	0.56	0.50	0.47	0.60	0.20	0.20	0.30
4,5-Dihydro-2-methyl-3(2 H)thiophenone	0.34	0.35	0.35	0.44	0.44	0.47	0.46	0.45	0.43
3-Octanone	0.13	0.13	0.12	0.11	0.11	0.12	0.08	0.09	0.09
2-Ethyl-6-methylpyrazine	0.42	0.44	0.41	0.16	0.15	0.18	0.14	0.14	0.19
2,2,4,6,6-Pentamethylheptane	0.18	0.19	0.19	0.16	0.15	0.17	0.06	0.07	0.12
β-Pinene P	0.62	0.61	0.64	0.66	0.62	0.68	0.54	0.55	0.69
n-Propyl benzene	0.65	0.71	0.66	0.36	0.34	0.49	0.25	0.28	0.54
Camphene	0.54	0.54	0.54	0.52	0.50	0.54	0.58	0.59	0.57
5-Methyl-3-heptanone	2.74	2.66	2.69	2.22	2.18	2.12	2.63	2.43	2.49
(R)-alpha-pinene	0.58	0.58	0.58	0.59	0.58	0.62	0.63	0.62	0.62
3-Methyl valeric acid	0.24	0.24	0.24	0.37	0.35	0.35	0.36	0.36	0.36
(E)-Hept-2-enal	0.46	0.57	0.45	0.15	0.13	0.28	0.08	0.10	0.29
Heptanal D	4.01	4.40	3.97	1.79	1.65	2.29	0.76	0.80	1.52
3-Heptanone D	0.65	0.63	0.67	0.73	0.76	0.77	0.77	0.75	0.75
1-Hexanol D	0.94	0.93	0.94	0.90	0.88	0.92	0.91	0.94	0.95
Ethyl 2-methylbutanoate	0.19	0.19	0.19	0.07	0.07	0.08	0.07	0.08	0.08
(E)-2-Hexenal D	1.31	1.34	1.36	0.37	0.35	0.37	0.29	0.29	0.32
2,5-Dimethylpyrazine	0.37	0.41	0.36	0.53	0.50	0.64	0.21	0.21	0.28
2,4-Dimethylheptane	0.10	0.10	0.10	0.13	0.13	0.15	0.17	0.17	0.18
Isobutyl propanoate	0.15	0.15	0.15	0.11	0.11	0.12	0.07	0.07	0.07
2-Hexanone	0.06	0.06	0.06	0.19	0.19	0.23	0.42	0.43	0.43
Hexanal D	9.25	9.40	9.28	5.84	5.75	5.88	2.80	2.76	3.17
Methyl 2-methyl butyrate	0.99	0.98	1.02	0.72	0.73	0.79	1.39	1.43	1.45
1-Pentanol D	0.35	0.34	0.35	0.31	0.30	0.30	0.11	0.12	0.13
Trans-2-pentenal D	0.15	0.16	0.16	0.14	0.13	0.14	0.04	0.04	0.04
2-Butanone 3-hydroxy D	1.73	1.69	1.73	7.07	6.63	6.74	3.99	4.00	3.90
n-pentanal D	2.56	2.58	2.53	0.84	0.84	0.93	0.32	0.35	0.54
3-Methylbutanol D	0.03	0.03	0.03	0.28	0.27	0.29	0.42	0.44	0.44
1 -Hydroxy-2-propanone	0.64	0.64	0.61	0.97	0.97	0.95	1.07	1.06	1.00
1-Heptene	0.26	0.26	0.25	0.28	0.28	0.29	0.25	0.24	0.28
2-Pentanone	0.23	0.22	0.23	0.28	0.27	0.30	0.26	0.25	0.28
1-Butanol D	4.99	5.03	5.06	2.10	2.21	2.27	2.12	2.13	2.29

Acetic acid	1.41	1.41	1.35	1.94	1.85	1.82	1.82	1.83	1.80
1-Propanol, 2-methyl	0.13	0.13	0.14	0.13	0.13	0.13	0.19	0.19	0.20
Ac. acetic ethyl ester D	2.32	2.21	2.11	3.05	3.03	2.94	3.74	3.78	3.52
Butanal	0.93	0.93	0.90	0.69	0.70	0.76	0.43	0.50	0.59
2,3-Butandione	0.58	0.63	0.58	0.69	0.72	0.79	0.56	0.56	0.73
2-Butanone D	2.06	2.25	2.09	1.31	1.40	1.62	0.76	0.77	1.18
n-Propyl acetate	0.17	0.18	0.17	0.10	0.12	0.12	0.14	0.11	0.13
2-Methylpropanal	0.12	0.13	0.16	0.10	0.12	0.12	0.13	0.11	0.16
1-Propanol	14.05	14.34	13.82	7.71	8.12	8.18	8.27	8.24	8.78
1-Penten-3-ol	0.35	0.36	0.36	0.25	0.24	0.24	0.10	0.11	0.13
Propanal	2.77	2.67	2.67	2.49	2.20	2.20	3.42	3.44	3.43
Hexanoic acid methyl ester	1.26	1.29	1.27	0.96	0.99	1.02	1.21	1.24	1.22
3-Methyl-2-butenal-D	1.49	1.47	1.46	1.88	1.88	1.88	1.04	1.05	1.10
4-methyl-3-Penten-2-one	0.82	0.80	0.80	0.85	0.86	0.83	0.56	0.58	0.59
2,3-Pentanedione	0.36	0.35	0.35	0.62	0.62	0.63	0.56	0.54	0.46
2-Pentanone, 3-methyl	0.02	0.01	0.01	0.02	0.02	0.03	0.04	0.04	0.04
3-Methylbutanoic acid	0.13	0.13	0.12	0.14	0.14	0.14	0.13	0.13	0.14
2-Pentylfuran	0.24	0.22	0.25	0.16	0.16	0.13	0.08	0.09	0.09
1,2-Dimethoxyethane	1.46	1.51	1.44	2.56	2.56	2.63	1.74	1.75	1.74
Hexanenitrile	0.05	0.05	0.05	0.05	0.04	0.05	0.04	0.04	0.03
Diethyl disulfide	0.22	0.21	0.23	0.21	0.21	0.22	0.21	0.21	0.20
2-Furanmethanol acetate	0.05	0.05	0.05	0.03	0.04	0.05	0.03	0.03	0.04

Table S2. 20 Years of Volatile Compounds Content

Compounds	gs14-1	gs14-2	gs14-3	gs21-1	gs21-2	gs21-3	gs22-1	gs22-2	gs22-3
Nerolidol	1.66	1.64	1.60	1.69	1.72	1.53	1.78	1.73	1.73
(E,E)-alpha-Farnesene	1.65	1.66	1.60	1.42	1.43	1.34	1.46	1.50	1.45
β-ionone	1.68	1.63	1.63	1.52	1.50	1.37	1.43	1.39	1.29
d-Longifolene	2.83	3.03	2.97	2.27	2.37	2.15	2.25	2.31	2.23
(-)-Carvone	0.59	0.59	0.57	0.43	0.42	0.36	0.43	0.44	0.40
Carveol	0.13	0.15	0.15	0.13	0.15	0.13	0.13	0.12	0.13
Linalool	0.46	0.46	0.45	0.16	0.14	0.14	0.26	0.26	0.26
(E)-2-Octenal	0.23	0.23	0.23	0.11	0.12	0.13	0.09	0.10	0.10
(+)-Limonene D	0.11	0.12	0.11	0.12	0.12	0.12	0.11	0.11	0.11
n-Butyl lactate	0.17	0.16	0.14	0.21	0.20	0.20	0.21	0.20	0.19
Acetic acid, hexyl ester	2.82	2.83	2.86	1.70	1.74	1.90	1.49	1.52	1.64
(Z)-3-Hexenyl acetate	2.27	2.34	2.29	0.52	0.54	0.67	0.48	0.50	0.64
2, 3, 5-Trimethylpyrazine	0.65	0.67	0.64	0.36	0.36	0.43	0.42	0.43	0.51
4,5-Dihydro-2-methyl-3(2 H)thiophenone	0.50	0.50	0.51	0.50	0.50	0.50	0.61	0.60	0.59
3-Octanone	0.15	0.15	0.16	0.11	0.10	0.10	0.12	0.11	0.13
2-Ethyl-6-methylpyrazine	0.28	0.28	0.30	0.14	0.13	0.14	0.16	0.16	0.17
2,2,4,6,6-Pentamethylheptane	0.16	0.17	0.18	0.09	0.09	0.12	0.06	0.05	0.07
β-Pinene P	0.64	0.68	0.71	0.64	0.63	0.66	0.61	0.60	0.62

n-Propyl benzene	0.54	0.56	0.54	0.27	0.28	0.42	0.24	0.25	0.33
Camphene	1.03	1.03	1.05	0.39	0.40	0.38	0.41	0.44	0.41
5-Methyl-3-heptanone	2.81	2.80	2.78	2.54	2.36	2.24	2.02	2.02	1.94
(R)-alpha-pinene	0.73	0.72	0.72	0.52	0.53	0.53	0.56	0.57	0.57
3-Methyl valeric acid	0.25	0.25	0.23	0.34	0.35	0.34	0.38	0.37	0.38
(E)-Hept-2-enal	0.35	0.36	0.39	0.10	0.10	0.19	0.09	0.09	0.13
Heptanal D	3.57	3.76	3.85	1.16	1.17	1.57	1.53	1.62	2.04
3-Heptanone D	0.74	0.77	0.77	0.78	0.75	0.75	0.63	0.56	0.58
1-Hexanol D	1.18	1.20	1.19	1.00	1.01	0.97	0.70	0.73	0.67
Ethyl 2-methylbutanoate	0.15	0.14	0.14	0.05	0.06	0.06	0.07	0.06	0.06
(E)-2-Hexenal D	1.03	1.02	1.03	0.22	0.21	0.24	0.26	0.29	0.28
2,5-Dimethylpyrazine	0.44	0.46	0.48	0.36	0.37	0.46	0.46	0.48	0.55
2,4-Dimethylheptane	0.18	0.19	0.19	0.16	0.17	0.16	0.22	0.21	0.21
Isobutyl propanoate	0.10	0.10	0.09	0.10	0.10	0.11	0.05	0.06	0.06
2-Hexanone	0.13	0.12	0.12	0.25	0.25	0.25	0.26	0.25	0.25
Hexanal D	7.97	7.85	7.89	4.40	4.39	4.38	2.90	2.88	3.01
Methyl 2-methyl butyrate	1.70	1.70	1.71	1.20	1.19	1.21	1.56	1.52	1.52
1-Pentanol D	0.25	0.25	0.27	0.21	0.21	0.19	0.12	0.13	0.12
Trans-2-pentenal D	0.14	0.14	0.14	0.05	0.05	0.06	0.06	0.07	0.07
2-Butanone 3-hydroxy D	2.21	2.22	2.21	3.68	3.70	3.56	4.74	4.84	4.67
n-pentanal D	1.39	1.43	1.48	0.69	0.66	0.75	0.23	0.23	0.27
3-Methylbutanol D	0.08	0.07	0.07	0.37	0.38	0.37	0.37	0.36	0.35
1 -Hydroxy-2-propanone	1.03	1.02	1.01	0.90	0.90	0.88	1.19	1.31	1.31
1-Heptene	0.27	0.27	0.26	0.26	0.26	0.28	0.25	0.25	0.27
2-Pentanone	0.23	0.22	0.22	0.31	0.31	0.32	0.91	0.89	0.87
1-Butanol D	3.16	3.21	3.18	2.53	2.55	2.56	1.49	1.40	1.49
Acetic acid	2.07	2.03	2.08	1.65	1.71	1.64	1.77	1.85	1.79
1-Propanol, 2-methyl	0.12	0.12	0.11	0.21	0.23	0.21	0.10	0.11	0.11
Ac. acetic ethyl ester D	3.97	3.97	4.04	3.53	3.53	3.41	8.38	8.21	8.21
Butanal	0.95	0.97	1.04	0.67	0.68	0.70	0.41	0.38	0.41
2,3-Butandione	0.67	0.72	0.75	0.63	0.62	0.74	0.95	0.92	1.07
2-Butanone D	1.65	1.73	1.82	1.21	1.18	1.42	0.95	0.89	0.95
n-Propyl acetate	0.09	0.10	0.10	0.17	0.18	0.18	0.16	0.15	0.14
2-Methylpropanal	0.07	0.09	0.09	0.10	0.11	0.12	0.05	0.06	0.07
1-Propanol	10.40	10.49	10.63	8.64	8.82	8.75	6.56	6.48	6.74
1-Penten-3-ol	0.33	0.33	0.32	0.16	0.17	0.17	0.07	0.07	0.07
Propanal	3.43	3.39	3.77	3.10	3.19	3.05	1.12	1.11	1.06
Hexanoic acid methyl ester	1.25	1.24	1.21	1.17	1.20	1.16	0.99	1.02	0.99
3-Methyl-2-butenal-D	1.70	1.65	1.67	1.36	1.39	1.35	1.98	2.01	2.04
4-methyl-3-Penten-2-one	0.87	0.85	0.83	0.66	0.67	0.70	0.62	0.62	0.63
2,3-Pentanedione	0.49	0.49	0.48	0.47	0.47	0.47	0.88	0.96	0.95
2-Pentanone, 3-methyl	0.02	0.02	0.02	0.04	0.04	0.04	0.03	0.03	0.03
3-Methylbutanoic acid	0.17	0.17	0.17	0.14	0.13	0.14	0.17	0.18	0.17
2-Pentylfuran	0.20	0.21	0.21	0.10	0.11	0.12	0.11	0.12	0.12
1,2-Dimethoxyethane	1.82	1.83	1.83	2.07	2.08	2.12	2.32	2.36	2.39
Hexanenitrile	0.06	0.05	0.06	0.04	0.04	0.04	0.05	0.05	0.04
Diethyl disulfide	0.21	0.21	0.20	0.19	0.19	0.20	0.14	0.13	0.13
2-Furanmethanol acetate	0.04	0.04	0.04	0.04	0.03	0.04	0.03	0.04	0.03

Table S3. 15 Years of ginsenosides Content

samples	15 years wild ginseng								
	gs10-1	gs10-2	gs10-3	gs18-1	gs18-2	gs18-3	gs19-1	gs19-2	gs19-3
Rg1	0.3358	0.338	0.3383	0.2492	0.2482	0.2489	0.2201	0.2173	0.2192
Re	0.595	0.5958	0.596	0.2756	0.2759	0.2752	0.2093	0.2082	0.2091
Rf	0.1048	0.1043	0.107	0.0358	0.0358	0.0356	0.028	0.0253	0.0268
Rb1	0.9245	0.9285	0.926	0.2436	0.2421	0.2431	0.2685	0.2666	0.2678
Rc	0.2565	0.27	0.269	0.1011	0.0992	0.1002	0.0619	0.06	0.0622
Rb2	0.2558	0.255	0.255	0.0993	0.0984	0.0987	0.056	0.0578	0.0573
Rd	0.0345	0.0378	0.033	0.0219	0.0233	0.0222	0.0128	0.0136	0.0128
Totalginsenosides	5.5512	5.5296	5.5361	3.7388	3.9759	3.8834	3.8031	3.8246	3.8132
Σ7 Ginsenosides	2.5069	2.5294	2.5243	1.0265	1.0229	1.0239	0.8566	0.8488	0.8552

Table S4. 15 Years of ginsenosides Content

samples	20years wild ginseng，								
	gs14-1	gs14-2	gs14-3	gs21-1	gs21-2	gs21-3	gs22-1	gs22-2	gs22-3
Rg1	0.70	0.70	0.69	0.41	0.40	0.41	0.33	0.33	0.33
Re	0.38	0.39	0.38	0.47	0.45	0.46	0.27	0.27	0.27
Rf	0.26	0.26	0.25	0.07	0.08	0.08	0.05	0.05	0.05
Rb1	0.95	0.94	0.94	0.60	0.60	0.60	0.33	0.33	0.33
Rc	0.40	0.39	0.39	0.15	0.15	0.15	0.13	0.13	0.13
Rb2	0.44	0.44	0.44	0.14	0.15	0.14	0.14	0.15	0.15
Rd	0.03	0.03	0.03	0.01	0.01	0.01	0.01	0.02	0.01
Totalginsenosides	5.05	5.03	5.04	5.79	5.83	5.81	4.45	4.47	4.46
Σ7 Ginsenosides	3.17	3.15	3.13	1.86	1.84	1.85	1.26	1.27	1.26
